# Resveratrol Targets Urokinase-Type Plasminogen Activator Receptor Expression to Overcome Cetuximab-Resistance in Oral Squamous Cell Carcinoma

**DOI:** 10.1038/s41598-019-48717-w

**Published:** 2019-08-21

**Authors:** Katsuhiro Uzawa, Antonio L. Amelio, Atsushi Kasamatsu, Tomoaki Saito, Akihiro Kita, Megumi Fukamachi, Yuki Sawai, Yuriko Toeda, Keitaro Eizuka, Fumihiko Hayashi, Ikuko Kato-Kase, Masataka Sunohara, Manabu Iyoda, Kazuyuki Koike, Dai Nakashima, Katsunori Ogawara, Yosuke Endo-Sakamoto, Masashi Shiiba, Yuichi Takiguchi, Mitsuo Yamauchi, Hideki Tanzawa

**Affiliations:** 1 Department of Oral Science, Graduate School of Medicine, Chiba University, 1-8-1 Inohana, Chuo-ku, Chiba 260-8670 Japan; 2 Department of Dentistry and Oral-Maxillofacial Surgery, Chiba University Hospital, 1-8-1 Inohana, Chuo-ku, Chiba 260-8677 Japan; 3 Division of Oral and Craniofacial Health Sciences, UNC Adams School of Dentistry, University of North Carolina at Chapel Hill, Chapel Hill, NC 27599-7455 USA; 4 Lineberger Comprehensive Cancer Center, UNC School of Medicine, University of North Carolina at Chapel Hill, Chapel Hill, NC 27599-7455 USA; 5 Biomedical Research Imaging Center, UNC School of Medicine, University of North Carolina at Chapel Hill, Chapel Hill, NC 27599-7455 USA; 6 Department of Anatomy, School of Life Dentistry at Tokyo, Nippon Dental University, 1-9-20 Fujimi, Chiyoda-ku, Tokyo 102-8159 Japan; 7 Department of Medical Oncology, Graduate School of Medicine, Chiba University, 1-8-1 Inohana, Chuo-ku, Chiba 260-8670 Japan

**Keywords:** Oral cancer, Oral cancer

## Abstract

Drug resistance to anti-cancer agents is a major concern regarding the successful treatment of malignant tumors. Recent studies have suggested that acquired resistance to anti-epidermal growth factor receptor (EGFR) therapies such as cetuximab are in part caused by genetic alterations in patients with oral squamous cell carcinoma (OSCC). However, the molecular mechanisms employed by other complementary pathways that govern resistance remain unclear. In the current study, we performed gene expression profiling combined with extensive molecular validation to explore alternative mechanisms driving cetuximab-resistance in OSCC cells. Among the genes identified, we discovered that a urokinase-type plasminogen activator receptor (uPAR)/integrin β1/Src/FAK signal circuit converges to regulate ERK1/2 phosphorylation and this pathway drives cetuximab-resistance in the absence of EGFR overexpression or acquired EGFR activating mutations. Notably, the polyphenolic phytoalexin resveratrol, inhibited uPAR expression and consequently the signaling molecules ERK1/2 downstream of EGFR thus revealing additive effects on promoting OSCC cetuximab-sensitivity *in vitro* and *in vivo*. The current findings indicate that uPAR expression plays a critical role in acquired cetuximab resistance of OSCC and that combination therapy with resveratrol may provide an attractive means for treating these patients.

## Introduction

Targeted molecular therapies inhibit specific regulators of oncogenic signaling cascades essential for development of malignant tumors^1^. Considerable evidence indicates that numerous genetic alterations contribute to tumorigenesis of human oral squamous cell carcinomas (OSCCs)^2,3^. This has prompted researchers to accelerate investigations aimed at creating targeted molecular therapies for this malignant tumor by exploiting therapeutic vurnerabilites in pathways that are dysregulated as a result of these genetic alterations. For example, expectations have changed regarding therapeutic strategies such as monoclonal antibodies and small-molecule kinase inhibitors that target the epidermal growth factor receptor (EGFR). Given that most OSCCs overexpress EGFR^4^, anti-EGFR antibodies, like cetuximab, are promising candidate therapeutic agents for human OSCCs. However, unlike conventional cisplatin-based chemotherapy, cetuximab does not have sustained effects due to emergence of acquired resistance caused by genetic alterations such as EGFR and KRAS gain-of-function mutations^5,6^. In addition, as reported by Stein-O’Brien *et al*.^7^, other dynamic molecular changes may be associated with drug-resistant phenotypes. Therefore, it is of clinical importance to identify potential novel pathway(s) that mediate this acquired resistance phenotype to achieve better responses to cetuximab. In fact, recent studies have shown that the expression status of a glutamine transporter, solute carrier 1 family member 5 (SLC1A5), in human colorectal cancer patients dictates their response to cetuximab regardless of EGFR/KRAS mutational status, thus highlighting the importance of currently unknown/unrecognized novel molecular pathways driving cetuximab resistance^8,9^. Moreover, inhibiting SLC1A5 significantly enhanced the inhibitory efficacy of cetuximab, suggesting that unknown molecular mechanism(s) may be involved in other human malignancies such as cetuximab-resistant SCC of the oral cavity.

In the current study, we generated three independent cetuximab-resistant OSCC cell lines that lack known hotspot *EGFR*/*KRAS* mutations. We then analyzed these cetuximab-resistant lines relative to their parental cetuximab-sensitive counterparts to search for novel molecular pathway(s) that govern drug resistance and whose targeting confers renewed sensitivity. Herein, we identified a urokinase-type plasminogen activator receptor (uPAR)/integrin β1/Src/FAK signaling circuit as a novel mechanism governing cetuximab-resistance in OSCC cells that can be effectively inhibited by resveratrol (a polyphenolic phytoalexin). These findings reveal an alternative strategy for re-sensitizing the cetuximab-resistance phenotype via blockade of this signaling pathway in OSCC.

## Materials and Methods

### Reagents

Cetuximab and resveratrol were purchased from Merck KGaA (Darmstadt, Germany). Cell Signaling Technology (Dancers, MA) provided the following antibodies: anti-EGFR (#4267), anti-Phospho-EGFR (#2234), anti-Integrinβ1 (#4706), anti-Src (#2108), anti-Phospho-Src Family (#2101), anti-FAK (#3285), anti-Phospho-FAK (#3283), anti-ERK1/2 (#4695), and anti-Phospho-ERK1/2 (#4370). Santa Cruz Biotechnology, Inc. (Dallas, TX) provided the antibodies against uPAR (sc-10815) and glyceraldehyde 3-phosphate dehydrogenase (GAPDH) (sc-3223).

### Cell lines

All human oral SCC-derived cell lines (SAS, Sa3, and HSC-3) were purchased from and authenticated by the Human Science Research Resources Bank or the RIKEN Bio Resource Center and cultured as described previously^10,11^. All cells used in this study were within 10 passages from thawing, were routinely tested for Mycoplasma contaminations using EZ-PCR Mycoplasma Test Kit (Biological Industries, Cromwell, CT).

To establish cetuximab-resistant cells, the parental cell lines (SAS-P, Sa3-P, and HSC-3-P) were exposed to varying concentrations of cetuximab (10, 20, 50, and 100 μg/mL for 24 hr) as described previously^12,13^. After the medium was replaced with normal medium for 7 days until the next mitosis, the cells were exposed to double-dosed of cetuximab for 24 hr. This process was performed successively until the final concentration of cetuximab reached 1,800 μg/mL. Genomic DNA was extracted from all cell lines studied, and they were assessed the known mutation status of the EGFR (exon 18 [G719X], exon 19 [E746_A750 deletion], exon 20 [V769_V774 insersions], exon 20 [T790M], and exon 21 [L858R]) and *KRAS* (codon 12/ 13) genes as described previously^14,15^.

### Gene expression profiling

RNA samples (50 ng) from each cell line, which passed quality control using the NanoDrop ND-1000 spectrophotometer (NanoDrop Technologies, Inc., Wilmington, DE), were analyzed by an Agilent SurePrint G3 Human GE microarray (Agilent Technologies, Santa Clara, CA). These microarray data have been reported in Gene Expression Omnibus (GEO) (https://www.ncbi.nlm.nih.gov/geo/) in under the reference number 114928. The expression intensity values of significantly differentially expressed genes were obtained by a fold-change cutoff greater than 2.0 or less than 0.5 (*P* < 0.01) and visualized by volcano plots^16^. Hierarchical clustering then was conducted using the genes that were shown to divide the cell lines into two groups (cetuximab-sensitive and cetuximab-resistant). The genes were considered to be associated significantly with the z-score at a false discovery rate exceeding a two-fold change. The biologic networks and pathway analyses for identified genes were performed using IPA, and the canonical pathways and Ingenuity Tox List tools were overlaid on the networks.

To refine the genes associated with cetuximab-resistance in OSCC, we compared our gene expression data to 3 independent gene expression datasets downloaded from GEO (accession numbers: GSE98812, GSE63916, and GSE32975).

### RT-qPCR

Total RNA was prepared using TRIZOL reagent (Invitrogen, Carlsbad, CA). RT-qPCR was performed as described previously^17–19^, with the following primers designed at the Universal Probe Library Assay Design Center (https://www.roche-applied-science.com/sis/rtpcr/upl/index.jsp?id=uplct_030000): *uPAR* (forward, 5′-ACACCACCAAATGCAACGA-3′; reverse, 5′-CCCCTTGCAGCTGTAACAC-3′); *ARRDC4:* (forward, 5′-GGAGGTGGAGTACCTGAACG-3′; reverse, 5′- AAATTCATGTTTTCCAGGCTGT-3′); *LEPROT:* (forward, 5′-TGTTGTTTCTGCCTTTGGATT-3′; reverse, 5′-GCAGGCTCCCCATTTGAT-3′); *PHF15:* (forward, 5′-GTTGGTCAGTCGTGTTTTAAAGAG-3′; reverse, 5′-TTCGCCTCTTCTCTTCCATC3′); *DDRGK1:* (forward, 5′-GTGGGCCTACGCACTCAG-3′; reverse, 5′-CCCGGTCGTCAATCACAC-3′); *HLA-A:* (forward, 5′-TTGAGAGCCTACCTGGATGG-3′; reverse, 5′-TGGTGGGTCATATGTGTCTTG-3′), and *LAMC2:* (forward, 5′-CTCAGCCCAACGACTAGACC-3′; reverse, 5′-TCACCTGTTGATTCCCAAGA-3′). The quantified values were normalized to the *GAPDH* gene, and the results are presented as relative values compared with the controls.

### Cellular viability assay

After treatment with cetuximab or resveratrol, the cellular viability was measured by the 3-(4,5-dimethylthiazol-2-yl)-5-(3-carboxymethoxyphenyl)-2-(4-sulfophenyl)-2H-tetrazolium (MTS) assay using CellTiter 96®AQueous Assay kit (Promega, Fitchburg, WI). Twenty microliters of the MTS reagent was added directly to the adherent cells, which were incubated at 37 °C for 24 hr. Absorbance then was recorded at 490 nm using a Benchmark Plus Microplate Reader (Bio-Rad, Philadelphia, PA). Each assay was repeated three times independently.

### Cellular proliferation assay

To evaluate the difference between cetuximab-resistant cells and the parental cell lines, we analyzed cellular growth. These cells were seeded in 6-cm dishes at a density of 1 × 10^4^ viable cells and cultured for 168 hours and counted every 24 hours. At the indicated time points, the cells were trypsinized and counted in triplicate using a hemocytometer.

### Immunoblotting

After the respective treatments, the cells were harvested and washed twice with phosphate buffered saline and centrifuged. Immunoblotting was performed as reported previously^20^ with each appropriate antibody already mentioned and visualized by exposing the membranes to ChemiDoc XRS Plus system and the signal intensities were quantified using the Image Lab system (Bio-Rad Laboratories)^11,21^. Densitometric uPAR protein data were normalized to GAPDH protein levels. (Bio-Rad Laboratories).

### Transfection with shRNA plasmid

To knock down uPAR expression in the resistance strains (SAS-R, Sa3-R, and HSC-3-R), they were transfected with shRNA targeting (shuPAR) and control shRNA (shMock) (Santa Cruz Biotechnology) using Lipofectamine 3000 (Invitrogen), according to the manufacturers’ instructions. The stable transfectants were isolated in a culture medium containing puromycin (2 µg/ml) (Santa Cruz Biotechnology). The efficiency of uPAR knockdown was assessed by RT-qPCR and immunoblotting.

### Murine experiments

Animal handling and all animal experiments followed the international guidelines. Specifically, to investigate whether uPAR expression contributed to cetuximab resistance and the antitumor activity of cetuximab combined with resveratrol, we used the SAS-R and Sa3-R cells for *in vivo* validation. The cells (1 × 10^6^ cells) were injected subcutaneously into the backs of the female athymic nude mice, the BALB/cAnNCrj-nu/nu strain (Oriental Yeast Co., Ltd., Andover, MA) as described previously^22^. The mice were subjected to different treatments, with three mice in each group: shMock + DMSO, shMock + cetuximab, shuPAR + DMSO, shuPAR + cetuximab, controls (DMSO), cetuximab, resveratrol, and cetuximab combined with resveratrol. The mice were treated with cetuximab (10 mg/kg intraperitoneally three times weekly) and/or resveratrol (100 mg/kg intraperitoneally daily). The body weight was measured at least every 2 weeks. The excised tissues were fixed in 10% formalin and embedded in paraffin followed by deparaffinization and hydration. The specimens were stained with appropriate antibodies such as anti-uPAR (1:50 dilution), anti-Integrinβ1 (1:250 dilution), anti-ERK1/2 (1:250 dilution), and anti-Phospho-ERK1/2 (1:250 dilution) at 4 °C. As a negative control, triplicate sections were immunostained without exposure to primary antibodies (Supplementary Fig. S4).

### Clinical subjects for immunohistochemical analysis (IHC)

Tissue samples from primay tumors and matched normal oral tissues were obtained from patients undergoing cetuximab therapy at the Chiba University Hospital. All patients had given written informed consent and were enrolled in institutional protocols approved by the Chiba University bioethics review committee (reference No. 562 (300)), which was in accordance with the Declaration of Helsinki and Good Clinical Practice. IHC for detecting uPAR was carried out with the same condition as mentioned above. The immunostaining images were quantified by the IHC Profiler (https://sourceforge.net/projects/ihcprofiler/, Source Forge) for IHC score as described previously^23^ (Supplementary Fig. S5).

### Statistical analysis

All data are expressed as the mean ± standard error of the mean. Statistical differences were analyzed by Welch’s t test (two group comparisons) or ANOVA (multiple group comparisons). *P*  < 0.05 was considered significant.

### Ethical approval

All protocols involving mice were approved by the ethical committee of the Graduate School of Medicine, Chiba University in accordance with relevant guidelines and regulations.

## Results

### mRNA profiling to identify genes related to cetuximab resistance in OSCC

**Figure 1:**
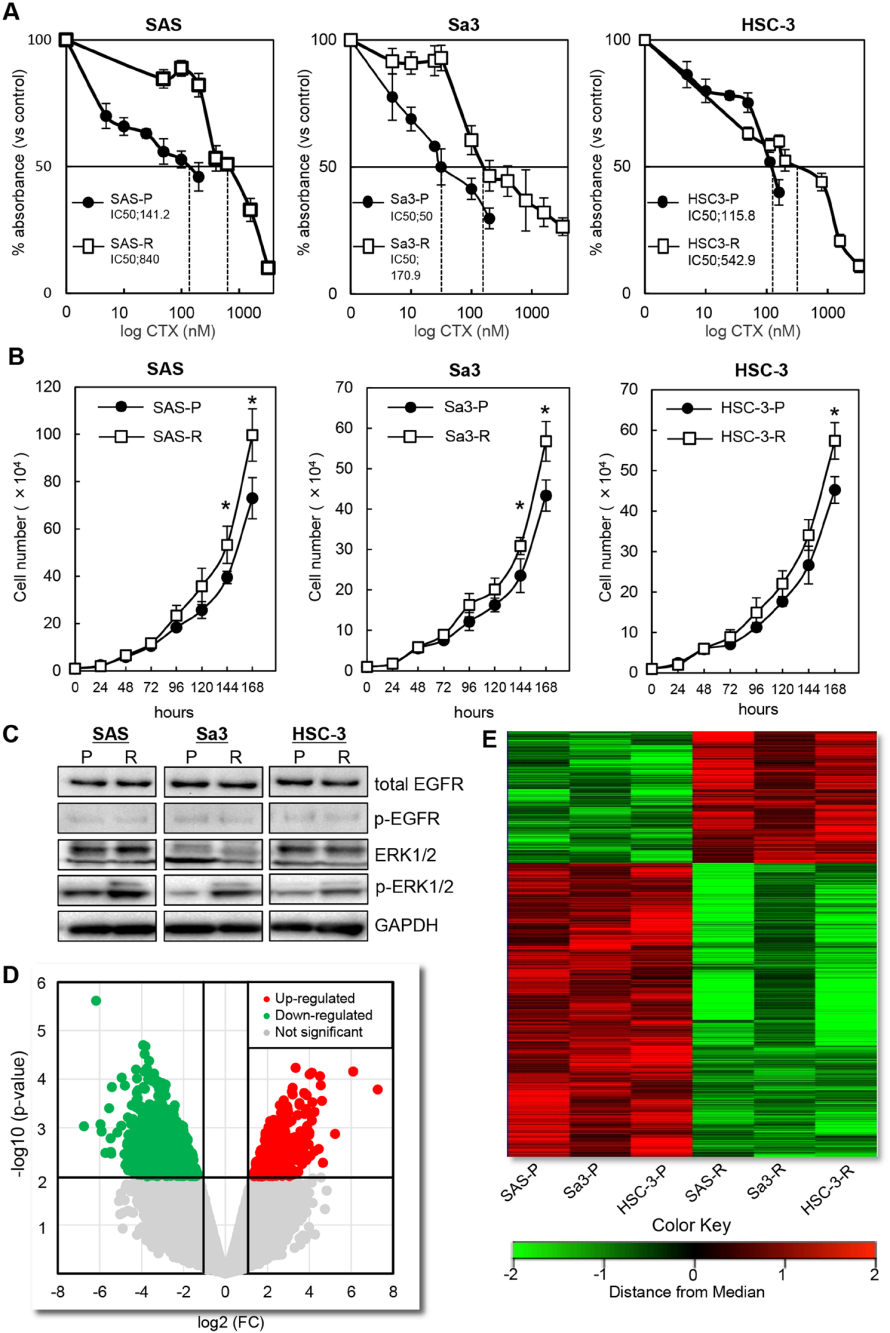
Characteristics and mRNA expression profilings for the cetuximab-resistant cells. (**A**) An MTS assay of the cetuximab-resistant cells and the parental cells in the presence of cetuximab at various concentrations (0–3200 nM) for 24 hr. More than a three-fold increase is seen in the IC50 value in cetuximab-resistant cells compared to parental cells. (**B**) Proliferation assay of the cetuximab-resistant cells and the parental cells without cetuximab. Cetuximab-resistant cells display significant increases in proliferative rates (*P < 0.05) compared to the parental cells at least after 7 days (168 hours) in culture. All graphs represent mean of three independent experiments ± SEM. (**C**) Increased phosphorylation of ERK1/2 in the cetuximab-resistant cells with cetuximab (100 nM) by immunoblot analysis. The expression levels of EGFR, phosphorylated EGFR (p-EGFR), and ERK1/2 do not change in any of the cell lines examined. P: parental cells, R: cetuximab-resistant cells. (**D**) Volcano plots of significant genes with *P* < 0.01 and more than a two-fold change or less than a 0.5-fold change expressed in 450 up-regulated genes and 992 down-regulated genes. The x-coordinate represents the (log2) fold-change (FC) and y represents the t-statistic or − log10 of the *P* value. (**E**) Heat maps of the 450 up-regulated genes or the 992 down-regulated genes in cetuximab-resistant cells. The red transcripts are up-regulated at least two-fold in cetuximab-resistant cells compared with the parental cells, and those in green are similarly down-regulated.

To determine the molecular mechanisms governing acquired resistance to cetuximab, we established three drug resistance cellular models by culturing OSCC-derived cell lines, including SAS, Sa3, and HSC-3, with increasing cetuximab concentrations. While the inhibitory concentration (IC) 50 values for cetuximab in parental cell lines were 141.2 ± 65.4 nM, 50.0 ± 24.1 nM, and 115.8 ± 17.5 nM for the SAS-P, Sa3-P, and HSC-3-P lines, respectively, the IC50 increased to 840.0 ± 91.7 nM, 170.9 ± 33.6 nM, and 542.9 ± 42.9 nM in the resistant cell lines (Fig. 1A). Ninety days later, these cetuximab-resistant cell lines, i.e., SAS-R, Sa3-R, and HSC-3-R, were cultured stably in cetuximab (12 nM)-containing medium, which confirmed a three-fold increase in the IC50 values in all cetuximab-resistant cells generated compared to the parental cells. Cells that were resistant to the anti-proliferative effects of cetuximab displayed an increased proliferative rate compared to untreated controls (Fig. 1B). Acquired resistance to cetuximab targeted therapy has been shown to occur via several mechanisms including EGFR overexpression, altered EGFR degradation, gain-of-function EGFR mutations, and/or gain-of-function mutations in downstream pathway effectors such as KRAS^5,6,24^. However, Sanger sequencing confirmed that all cell lines lacked any nucleotide variations in *EGFR* and *KRAS* regardless of cetuximab sensitivity (Supplementary Fig. S1). Intriguingly, while the cetuximab-resistant SAS-R, Sa3-R, and HSC-3-R cell lines showed no significant change in total EGFR, phosphorylated EGFR (p-EGFR), or total ERK1/2 protein expression, immunoblotting revealed elevated p-ERK1/2 levels in all cetuximab-resistant cell lines (treated with 100 nM of cetuximab) compared to each parental cell line (Fig. 1C). These results suggest that alternative pathway(s) for cetuximab-resistance exist that converge on downstream ERK1/2 signaling.

To elucidate the mechanisms responsible for emergence of acquired drug resistance, we performed gene expression analyses on microarray data collected for the parental cell lines relative to their cetuximab-resistant counterparts. Volcano plots show that 450 up-regulated genes and 992 down-regulated genes were significantly changed (*P* < 0.01) more than two-fold (Fig. 1D). Unsupervised hierarchical clustering of all differentially expressed genes showed clear cetuximab resistance-related gene expression patterns (Fig. 1E). Moreover, based on the common differentially expressed genes with more than a two-fold z-score (Supplementary Table S1), we selected the top 20 up-regulated genes for further validation by reverse transcriptase quantitative polymerase chain reaction (RT-qPCR) and confirmed eight commonly up-regulated genes, including *uPAR*, *ARRDC4*, *LEPORT*, *PHF15*, *DDRGK1*, *HLA-A*, *LAMC2*, and CD44 (Supplementary Fig. S3). Notably, 12 genes including *uPAR* formed part of a common overlapping cetuximab-resistance gene signature when we cross-examined several independent gene expression datasets by Hatakeyama *et al*.^14^ and performed this comparison with our up-regulated gene list (900 genes) relative to parental cetuximab-sensitive cells (Supplementary Fig. S2 and Table S1). Interestingly, uPAR is the only gene within this 12-gene signature previously shown to regulate an EGFR/p-ERK1/2-related signal pathway^25^, therefore, we ultimately selected uPAR for further *in vitro* and *in vivo* validation based on these data and Ingenuity Pathway Analysis (IPA) analysis.

### Effects of uPAR inhibition *in vitro*

**Figure 2:**
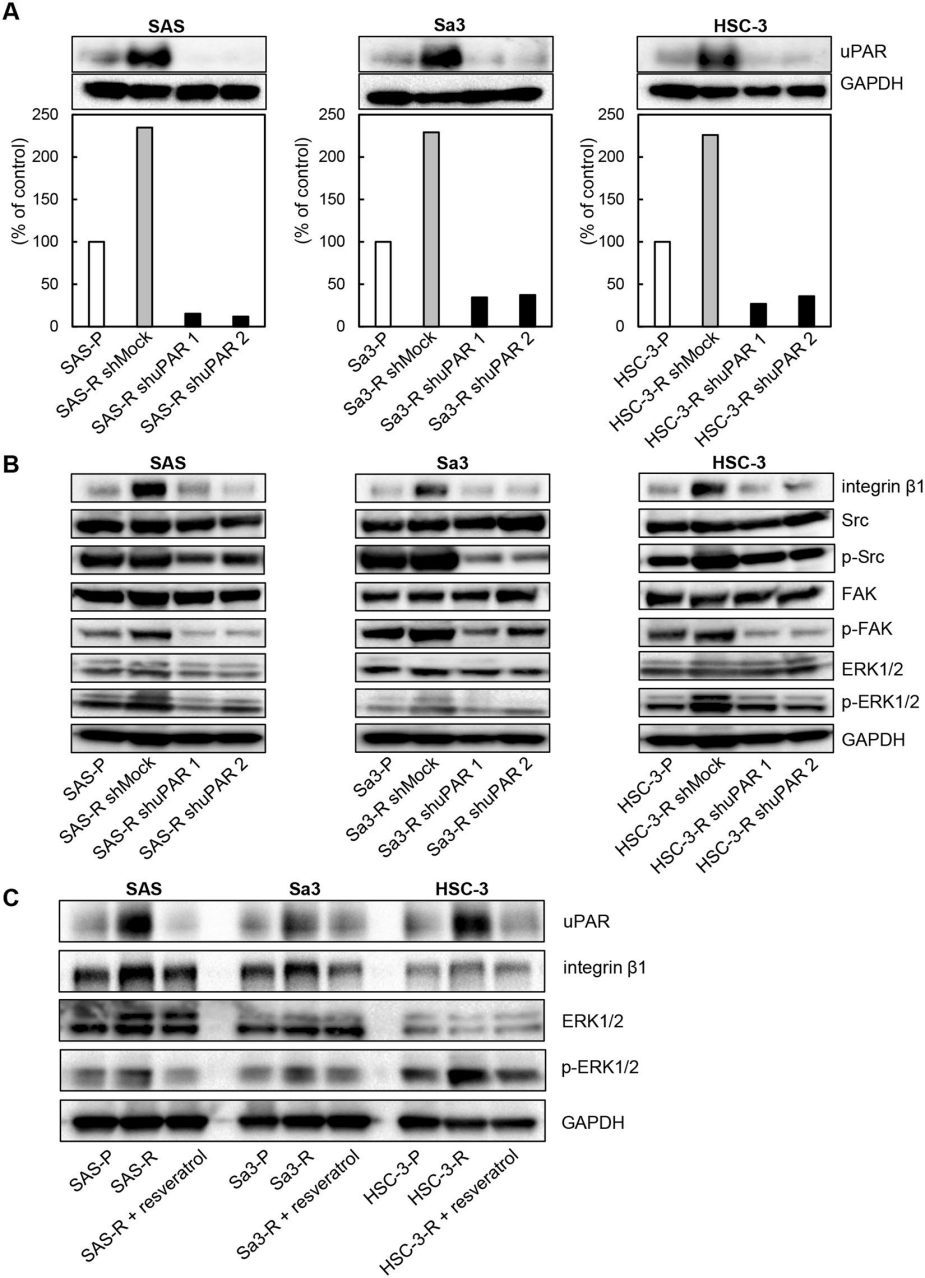
Effects of uPAR in cetuximab-resistance cell*s in vitro*. (**A**) Establishment of uPAR knockdown cells. Expression levels of uPAR in the cetuximab-resistant shuPAR cells are down-regulated compared to shMock cells. (**B**) The expression of uPAR-related genes in the cells treated with cetuximab (100 nM) for 24 hr. While integrin β1, p-Src, p-FAK, and p-ERK1/2 expression levels are up-regulation in the cetuximab-resistant shMock cells compared with the parental cells, and they are down-regulated in the shuPAR cells compared to the shMock cells. The total Src, FAK, and ERK1/2 protein levels remain unchanged in all cell lines examined. (**C**) The effects of resveratrol on the cetuximab-resistant cells. While uPAR, integrin β1, and p-ERK1/2 are down-regulated significantly in the presence of cetuximab (100 nM)/resveratrol (20 µM) in cetuximab-resistant cells, the steady-state level of total ERK remains constant in all cells under all conditions tested.

The observed significant increase in *uPAR* expression suggests a direct role in mediating cetuximab resistance. To test whether acquired resistance to cetuximab requires *uPAR*, we performed genetic knockdown using validated shRNA molecules specific to uPAR (Fig. 2A). Immunoblot analysis showed that targeted silencing by shuPAR knockdown significantly down-regulated uPAR levels (Fig. 2A). Moreover, downstream signaling pathways, such as integrin β1, p-Src, p-FAK, and p-ERK1/2 were all reduced in shuPAR treated but not in the shMock-treated cetuximab-resistant cell lines (Fig. 2B).

**Figure 3:**
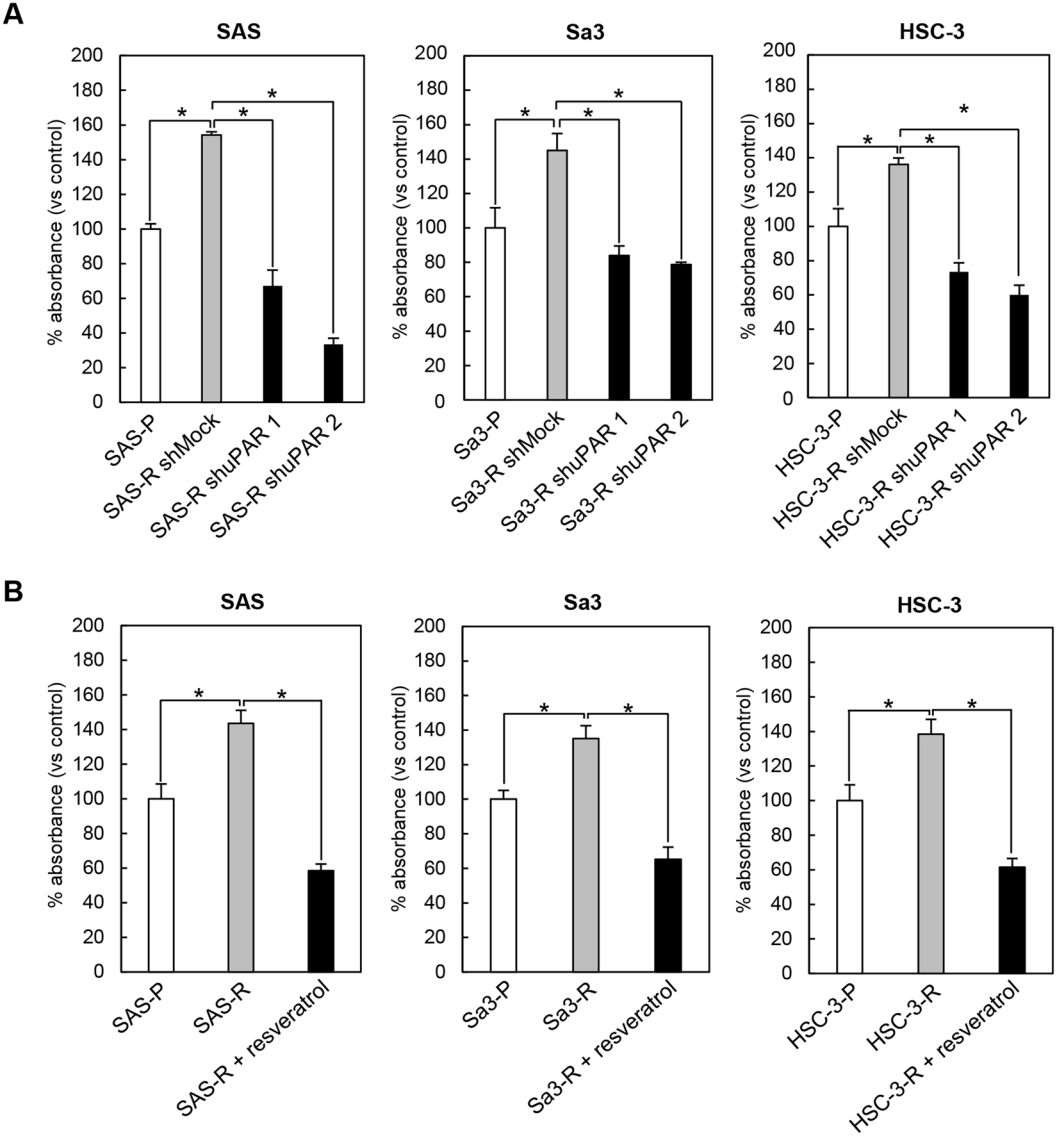
Analysis of oral SCC cells viability under the conditions of (A) shuPAR and (**B**) resveratrol treatment. The cellular viability measured by the MTS assay shows a significant reduction in cellular viability in the cetuximab-resistant shuPAR cells. Absorbance was measured at 490 nm. Data are expressed as the mean and standard deviation. All graphs represent mean of three independent experiments ± SEM. Asterisks indicate a significance difference (*P < 0.05).

To investigate the effect of *uPAR* knockdown via a pharmacologic agent on cetuximab-resistant OSCC cells, we chose to focus on resveratrol (3,4′,5-trihydroxystilbene), a naturally occurring compound with anti-cancer effects^26–31^ found in a variety of fruits and plants^32^ that has previously been shown to potently downregulate uPAR expression, as a therapeutic strategy for targeting cetuximab-resistant OSCC cells. Similar to the shuPAR condition, resveratrol-treated cetuximab-resistant cell lines expressed similar levels of total ERK1/2 compared to the parental cell lines, however, resveratrol treatment lead to a significant decrease in pERK1/2 levels and a robust downregulation of integrin β1 and uPAR was observed (Fig. 2C). We also found that both shRNA knockdown of *uPAR* or resveratrol treatment lead to a significant reduction of cellular viability in all cetuximab-resistant cell lines examined (Fig. 3A,B). There was no additional effect of combining shRNA knockdown of uPAR and resveratrol on cellular viability and ERK1/2 phosphorylation of SAS-R against cetuximab (Supplementary Fig. S6). This data suggest that the uPAR-dependent pathway is a major pathway that regulates resensitizing the cetuximab-resistant phenotype by resveratrol in SAS-R cells.

### Effects of uPAR inhibition *in vivo*

**Figure 4:**
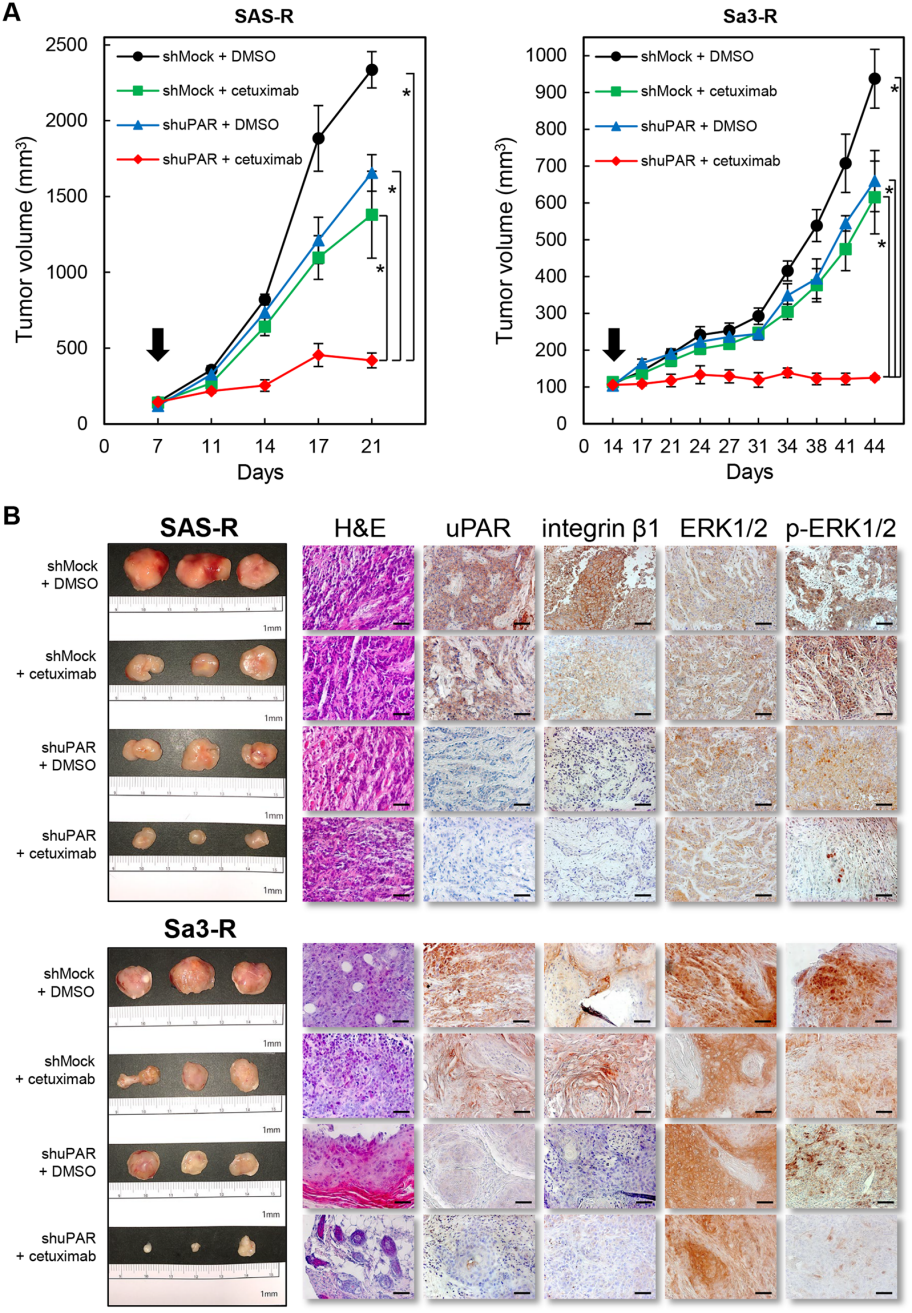
The effects of uPAR in cetuximab-resistant cells *in viv*o. (**A**) The tumor volume was calculated twice weekly after the injection. when the volume of the transplantation tumor reached 100 mm^3^ (arrows), the mice were treated with different drugs as indicated below (n = 3 per group). Significant antitumor growth activity by uPAR knockdown (shuPAR) combined with cetuximab is evident in SAS-R and Sa3-R cell xenografts. *P < 0.05. (**B**) The tumoral tissues from the shMock + DMSO, shMock + cetuximab, shuPAR + DMSO, and shuPAR + cetuximab were fixed in 10% formalin, and paraffin sections were prepared for hematoxylin and eosin (H&E) staining and immunohistochemistry (uPAR, integrin β1, ERK1/2, and p-ERK1/2). The scale bar indicates 50 μm.

**Figure 5:**
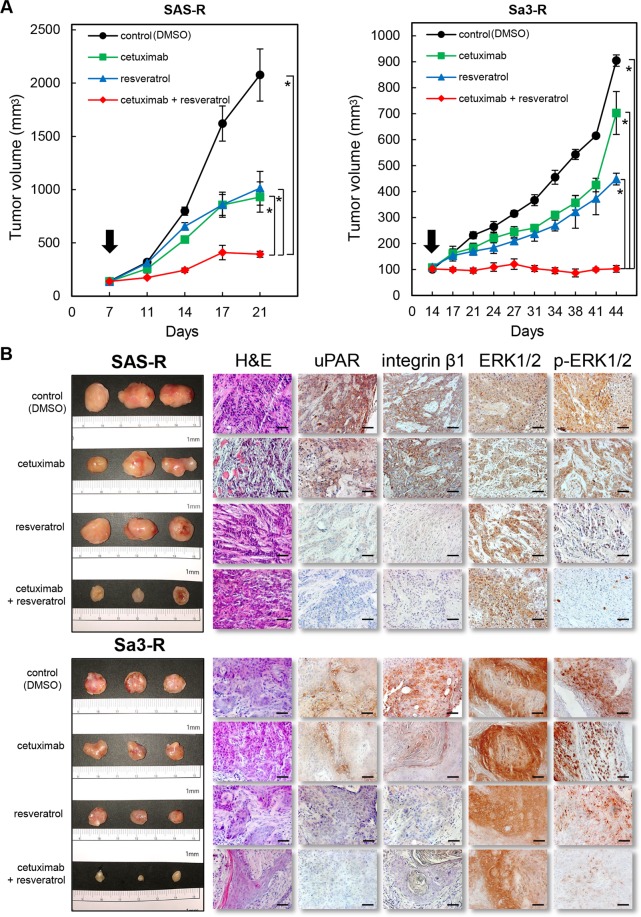
Effects of resveratrol in cetuximab-resistant cells *in vivo*. (**A**) The tumor volume was calculated twice weekly after the injection. when the volume of the transplantation tumor reached 100 mm^3^ (arrows), the mice were treated with different drugs as indicated below (n = 3 per group). Significant antitumor growth activity by resveratrol combined with cetuximab is evident in SAS-R and Sa3-R cell xenografts. *P < 0.05. (**B**) The tumoral tissues from the controls (DMSO), cetuximab, resveratrol, and cetuximab combined with resveratrol were fixed in 10% formalin, and paraffin sections were prepared for hematoxylin and eosin (H&E) staining and immunohistochemistry (uPAR, integrin β1, ERK1/2, and p-ERK1/2). Scale bars indicate 50 μm.

We next examined the effects of *uPAR* knockdown in combination with EGFR inhibition on tumor growth *in vivo*. As expected, conditions that combined cetuximab treatment with shuPAR displayed dramatic and synergistic antitumor activity, with growth inhibition levels of about 82.0% of the SAS-R cells at day 21 and 86.6% of the Sa3-R cells at day 44 (Fig. 4A). However, we did not see adequate antitumor growth inhibition in the SAS-R and Sa3-R cells treated with cetuximab or shuPAR alone (Fig. 4A). Immunohistochemical analyses showed dramatic differences in the expression status of uPAR, integrin β1, and p-ERK1/2 of the resected xenograft tumors. More importantly, the combination of cetuximab and shuPAR was more efficient than cetuximab alone or shuPAR alone in the down-regulation of protein expression levels of integrin β1 and uPAR and displayed a robust downregulation of pERK1/2 levels without affecting total ERK1/2 protien levels. (Fig. 4B, Supplementaly Fig. S5A). Consistently, and similar to the effect of uPAR depletion with shRNA *in vivo*, resveratrol significantly suppressed tumor growth and down-regulated proteins downstream of uPAR (Fig. 5A,B, and Supplementaly Fig. S5B). We also found that the average body weight of the resveratrol-treated mice never dropped lower than that of the control group at any time point (Supplementary Fig. S7).

### The uPAR expression level in OSCC tissues with or without cetuximab resistance

We next performed IHC to assess uPAR protein levels in primary OSCCs obtained from cetuximab treated patients. We observed significant and intense uPAR immunoreactivity in all human oral SCC tissues examined compared to the corresponding normal tissues. More importantly, uPAR protein expression levels in cetuximab-resistant OSCCs was much higher than that for cetuximab-sensitive cases (Supplementary Fig. S8). Collectively, these data indicate that up-regulating uPAR gene expression confers acquired cetuximab resistance in OSCC and that the uPAR inhibitor resveratrol is capable of restoring drug sensitivity to cetuximab.

## Discussion

Pharmacologic targeting of EGFR with the specific monoclonal antibody cetuximab is now a protagonist of molecular targeted therapy for recurrent/metastatic SCCs of the oral cavity^33^. However, its clinical use is limited due to acquired drug resistance. A few previous studies have shown that activation of the EGFR-dependent pathways is regarded as the main cause of drug resistance^9,34,35^.

**Figure 6:**
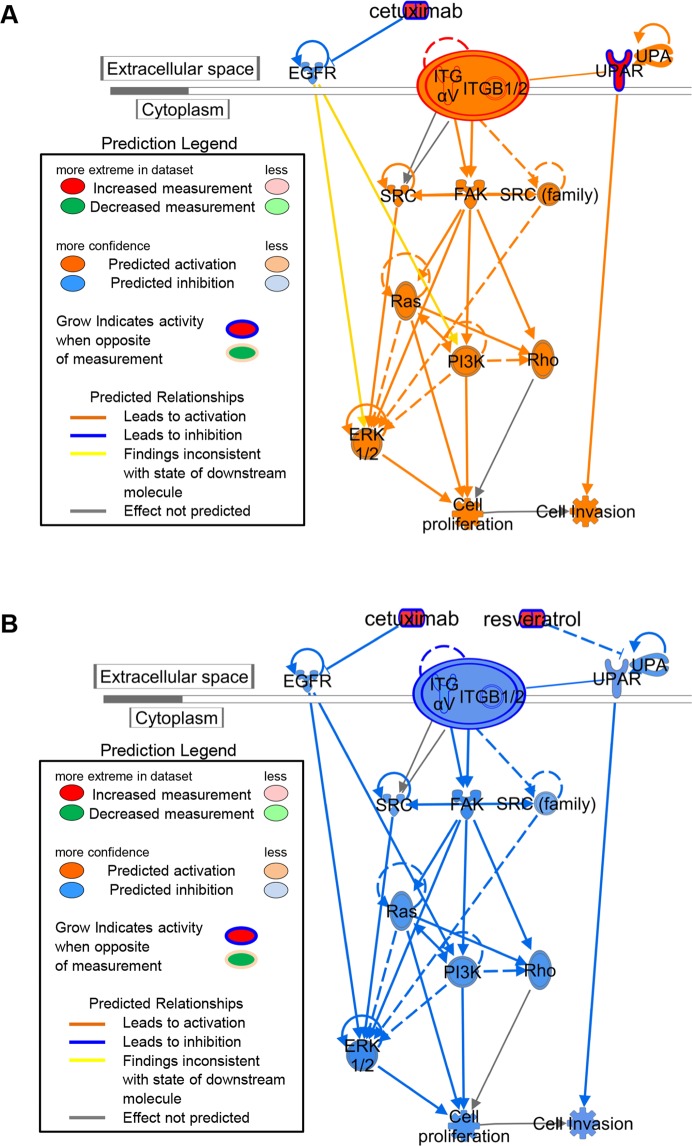
Ingenuity Pathway Analysis (IPA) of the canonical pathways for the cetuximab-resistant cells based on (**A**) increased expression of uPAR and (**B**) the predicted signaling pathway for uPAR inhibition by resveratrol leading to suppression of cetuximab-resistance in cancer cells. The pathways analyses were generated through the use of IPA (QIAGEN Inc., https://www.qiagenbioinformatics.com/products/ingenuity-pathway-analysis).

In the current study, we identified gene expression changes common to cetuximab-resistant cell lines and validated select up-regulated targets. Among 1,448 differentially expressed genes identified, we found that the expression levels of a small subset of candidate genes increased significantly in the cetuximab-resistant cells (Supplementary Table S1). Moreover, coupled with available gene expression data published by Hatakeyama *et al*.^14^, we have characterized *uPAR* as the only molecule reported to be a potential regulator of the EGFR-dependent signals^25^. Given that *uPAR* up-regulation has been detected in the primary OSCC tumors from patients with cetuximab resistance (Supplementary Fig. S8), we sought to test the hypothesis that cetuximab-resistance may be associated with uPAR and its downstream signal pathways. The IPA analysis (Fig. 6A) indicated that uPAR regulates an integrin β1 signaling axis that converges with activated EGFR pathways at the ERK1/2 siganling node. Our results reveal that both signals are involved in the acquired cetuximab-resistance mechanism, and at the same time, the effect of cetuximab is expected to be enhanced if these signals are controlled (Fig. 6B). Indeed, sphere-forming head and neck SCC cells possibly resistant to molecular targeting were insensitive to both anti-integrin β1 and anti-EGFR inhibitory antibodies^36^. In human pancreatic ductal adenocarcinoma cells, active integrin β1 is associated closely with primary resistance to cetuximab^37^. In addition, owing to the frequent aberrant expression levels of integrin β1 and EGFR in human cancer, previous studies have indicated that the integrin β1-EGFR signal pathway is the targeted option that can efficiently reduce tumor radioresistance^38–40^. Although it is apparent that multiple strategies for cetuximab-resistance have been assumed in human cancers, the above-mentioned observations and our results showed that active uPAR/integrin β1 signaling in cetuximab resistant OSCC cell lines (Figs 2B and 6A) supports our hypothesis that this pathway may play an alternative role in OSCC refractory to cetuximab treatment.

The next step in translational research is identification of highly selective chemical inhibitors for uPAR in cetuximab-resistant cell lines. Similar to the shuPAR effect, the combination of resveratrol and cetuximab led to almost complete suppression of tumor growth in mice resistant to cetuximab (SAS-R, Sa3-R) with significant down-regulation of the uPAR/integrin β1 signaling pathway (Figs 5A,B and 6B). Resveratrol, a multifunctional natural compound, has a variety of beneficial biologic activities in metabolic and neurodegenerative diseases and cardioprotection^41–43^.

Intriguingly, recent accumulated evidence shows that resveratrol inhibits specific human carcinogenesis^44^. Considering the impact of this anti-oncogenic activation, more concerted efforts are required toward finding a chemosensitization effect for resveratrol. Previous studies have reported a chemosensitizing effect of resveratrol in combined treatments with several anti-cancer drugs, such as docetaxel, cisplatin, 5-fluorouracyl, and oxaliplatin^45–49^. It is noteworthy that resveratrol induces down-regulation of *uPAR* and exerts an anti-proliferative effect on malignant tumor cells^27,28^. The current study found that uPAR was highly expressed in the resistant cell lines compared to the parental strains (Fig. 2A, and Supplementaly Table S1), suggesting that resveratrol may be more effective for treating patients refractory to cetuximab. Importantly, the combination of resveratrol and cetuximab significantly inhibited the growth of cetuximab-resistant tumor xenografts in nude mice without inducing systemic toxicity compared to resveratrol or cetuximab alone, and lead to a significant down-regulation of uPAR/integrinβ1/p-ERK1/2 (Fig. 5A,B), indicating that this combination may inhibit tumor growth in mice by suppressing two independent but convergent pathways (EGFR-dependent and uPAR-dependent pathways) simultaneously (Fig. 6B). Moreover, these findings suggest that the combination of resveratrol and cetuximab will be generalizable to all OSCC that acquire cetuximab resistance, either by EGFR overexpression or through activating mutations, since the uPAR forms part of a common downstream pathway that regulates ERK1/2 signaling. While no data exist for cetuximab-resistant OSCC cell lines that harbor mutant EGFR/KRAS, Hatakeyama *et al*.^14^ reported up-regulation of uPAR in cetuximab-resistant SCC1 cells that corresponds with increased phosphorylated EGFR in the presence of cetuximab.

Based on our findings and the canonical pathways outlined in Fig. 6, we speculate that cetuximab-resistance is mediated by uPAR upregulation regardless of EGFR mutational status. Thus, resveratrol may be effective for both EGFR mutant and wild-type OSCC cells. In fact, considerable evidence has shown widespread overexpression of uPAR in many cancers from disparate anatomic sites including the colon, lung, liver, breast, prostate, pancreas, kidney, bladder, and thyroid^50^. Therefore, this combination therapy might be expanded for use in additional other human malignancies that are refractory to anti-EGFR therapies.

Consequently, our preclinical *in vitro* and *in vivo* data may provide insights into additional and alternative treatments for cetuximab-resistant OSCC. Although the sample size for the clinical tumor tissues is relatively small, it will be important to design clinical studies to assess the prognostic value of increasing *uPAR* expression in patients with OSCC based on the status of *uPAR* expression in primary tumors (Supplementary Fig. S8), since cetuximab-treated OSCC patients have traditionally been divided into two groups (i.e., those with and without cetuximab resistance). Finally, future work focusing on combined treatment using an inhibitor of uPAR expression combined with cetuximab is needed to advance our understanding of drug resistance for other EGFR-targeted drugs, such as panitumumab, erlotinib, and gefitinib.

## Supplementary information

Supplementary Information

Supplementary Table S1

## Data Availability

All data generated or analyzed during this study are included in this published article. The datasets generated during and/or analysed during the current study are available from the corresponding author on reasonable request.
