# Supplementary material for: Resveratrol Targets Urokinase-Type Plasminogen Activator Receptor Expression to Overcome Cetuximab-Resistance in Oral Squamous Cell Carcinoma
Source: Sci Rep. 2019 Aug 21;9:12179. doi: 10.1038/s41598-019-48717-w (PMC6704133; doi:10.1038/s41598-019-48717-w)

Supplementary information for

Resveratrol Targets Urokinase-Type Plasminogen Activator Receptor Expression to Overcome Cetuximab-Resistance in Oral Squamous Cell Carcinoma

Katsuhiro Uzawa[1,2]*, Antonio L Amelio[3,4,5]*, Atsushi Kasamatsu[2], Tomoaki Saito[2], Akihiro Kita[2], Megumi Fukamachi[1], Yuki Sawai[1], Yuriko Toeda[1], Keitaro Eizuka[1], Fumihiko Hayashi[1], Ikuko Kase-Kato[1], Masataka Sunohara[6], Manabu Iyoda[2], Kazuyuki Koike[2], Dai Nakashima[2], Katsunori Ogawara[1], Yosuke Sakamoto-Endo[2], Masashi Shiiba[7], Yuichi Takiguchi[7], Mitsuo Yamauchi[3] and Hideki Tanzawa[1,2]

[1] Department of Oral Science, Graduate School of Medicine, Chiba University, 1-8-1 Inohana, Chuo-ku, Chiba 260-8670, Japan
[2] Department of Dentistry and Oral-Maxillofacial Surgery, Chiba University Hospital, 1-8-1 Inohana, Chuo-ku, Chiba 260-8677, Japan
[3] Division of Oral and Craniofacial Health Sciences, UNC Adams School of Dentistry, University of North Carolina at Chapel Hill, Chapel Hill, NC 27599-7455, USA
[4] Lineberger Comprehensive Cancer Center, University of North Carolina at Chapel Hill, Chapel Hill, NC 27599-7455, USA
[5] Biomedical Research Imaging Center, University of North Carolina at Chapel Hill, Chapel Hill, NC 27599-7455, USA
[6] Department of Anatomy, School of Life Dentistry at Tokyo, Nippon Dental University, 1-9-20 Fujimi, Chiyoda-ku, Tokyo 102-8159, Japan
[7] Department of Medical Oncology, Graduate School of Medicine, Chiba University, 1-8-1 Inohana, Chuo-ku, Chiba 260-8670, Japan

***Corresponding Authors:**
Katsuhiro Uzawa, DDS, PhD, FIBCSOMS
Editorial Board Member of Scientific Reports (Cancer Category)
Department of Oral Science
Graduate School of Medicine, Chiba University
1-8-1 Inohana, Chuo-ku
Chiba, 260-8670, Japan
Phone: +81-43-226-2300
E-mail: uzawak@faculty.chiba-u.jp

Antonio L. Amelio, PhD
Department of Oral and Craniofacial Health Sciences
Lineberger Comprehensive Cancer Center
University of North Carolina at Chapel Hill
Chapel Hill, NC 27599-7455, USA
Phone: (919) 537-3309
E-mail: alamelio@email.unc.edu

Supplementary Figure S1
Representative chromatographs of Sanger sequencing results for EGFR and KRAS where hotspot activating mutations are known to occur. Note that all cell lines lacked any nucleotide variations in EGFR and KRAS regardless of cetuximab sensitivity. P: parental cell lines, R: cetuximab-resistant cell lines.

Supplementary Figure S2
Cross-dataset comparison of gene sets (450 vs 900 genes) significantly correlated with cetuximab resistance in OSCC cell lines reveals 12 overlapped genes.

Supplementary Figure S3
RT-qPCR analysis for uPAR-related molecules. All gene expression levels analyzed were elevated in cetuximab-resistant cells when compared to each parental cells. All graphs represent mean of three independent experiments ± SEM, *$P < 0.05$.

Supplementary Figure S4
Negative control of figure 4B and Figure 5B. Sections were immunostained without exposure to primary antibodies.

Supplementary Figure S5
Immunohistochemical analyses shown in **(A)** Fig. 4B and **(B)** Fig. 5B were quantified using the IHC scoring system. The mean percentages of positive tumor cells were determined in three random fields in each section. The intensities of the stained cells were classified into four levels by the IHC Profiler (https://sourceforge.net/projects/ihcprofiler/, Source Forge). The cellular numbers and the staining intensities were multiplied to produce uPAR, integrin β1, ERK1/2, and p-ERK1/2 IHC scores. *$P < 0.05$.

Supplementary Figure S6
Analysis of cell viability and immunoblotting after treatment with a selective inhibitor of uPAR (cenupatide) in the presence of cetuximab. Cenupatide (#1006388-38-0) was purchased from Medkoo Biosciences, Inc. (NC, USA). Since some studies have shown that the Ki of cenupatide ranges from 1 to 100nM, we used cenupatide at a concentration of 10nM for 24 hours. (lanes 1 and 2) The cell viability/p-ERK expression status after treatment with cetuximab (100nM) did not decrease compared to non-treated SAS-R cells. (lanes 3 and 4) The cell viability/p-ERK expression status after treatment of cenupatide (10nM, 24h) have not seen a significant difference with/without resveratrol (20μM, 24h) under the condition of cetuximab (100nM) in SAS-R cells. (lane 5) The cell viability/p-ERK expression status after treatment with resveratrol (20μM, 24h) decreased compared to no-treated SAS-R cells. These data indicate that inhibition of uPAR is the major driver of resveratrols anti-tumor effects towards the cetuximab-resistant OSCCs cell line. N.S., no significant difference.

Supplementary Figure S7
The body weight of the mice was measured throughout the days of treatment. Cetuximab and resveratrol do not affect the body weight of the mice compared with the control group.

Supplementary Figure S8

Immunohistochemical analysis of uPAR in primary human OSCC tissue **(A)** and quantification of uPAR expression status **(B)**. Note that elevation of uPAR level was clearly evident in primary tumor tissues obtained from the cetuximab-resistant patients, when compared to the responders. Tumors were evaluated by RECIST v1.1 as follows; CR: complete response, PR: partial response, SD: stable disease, and PD: progressive disease, respectively. Scale bars indicate 50 μm.

Supplementary Figure S9

Full-length blots of Figure 1C

The samples derive from the same experiment and that gels/blots were processed in parallel. All blots were detected by the same exposure time and contrast.

Supplementary Figure S10

Full-length blots of Figure 2A

The samples derive from the same experiment and that gels/blots were processed in parallel. All blots were detected by the same exposure time and contrast.

Supplementary Figure S11

Full-length blots of Figure 2B (SAS)

The samples derive from the same experiment and that gels/blots were processed in parallel. All blots were detected by the same exposure time and contrast.

Supplementary Figure S12

Full-length blots of Figure 2B (Sa3)

The samples derive from the same experiment and that gels/blots were processed in parallel. All blots were detected by the same exposure time and contrast.

Supplementary Figure S13

Full-length blots of Figure 2B (HSC-3)

The samples derive from the same experiment and that gels/blots were processed in parallel. All blots were detected by the same exposure time and contrast.

Supplementary Figure S14

Full-length blots of Figure 2C

The samples derive from the same experiment and that gels/blots were processed in parallel. All blots were detected by the same exposure time and contrast.

Supplementary Figure S15

Immunoblotting analysis for cetuximab R and R-shMock cells.

The samples derive from the same experiment and that gels/blots were processed in parallel. All blots were detected by the same exposure time and contrast.

Supplementary Figure S16

Full-length blots of Supplementary Figure S6

The samples derive from the same experiment and that gels/blots were processed in parallel. All blots were detected by the same exposure time and contrast.

Supplementary Figure S1

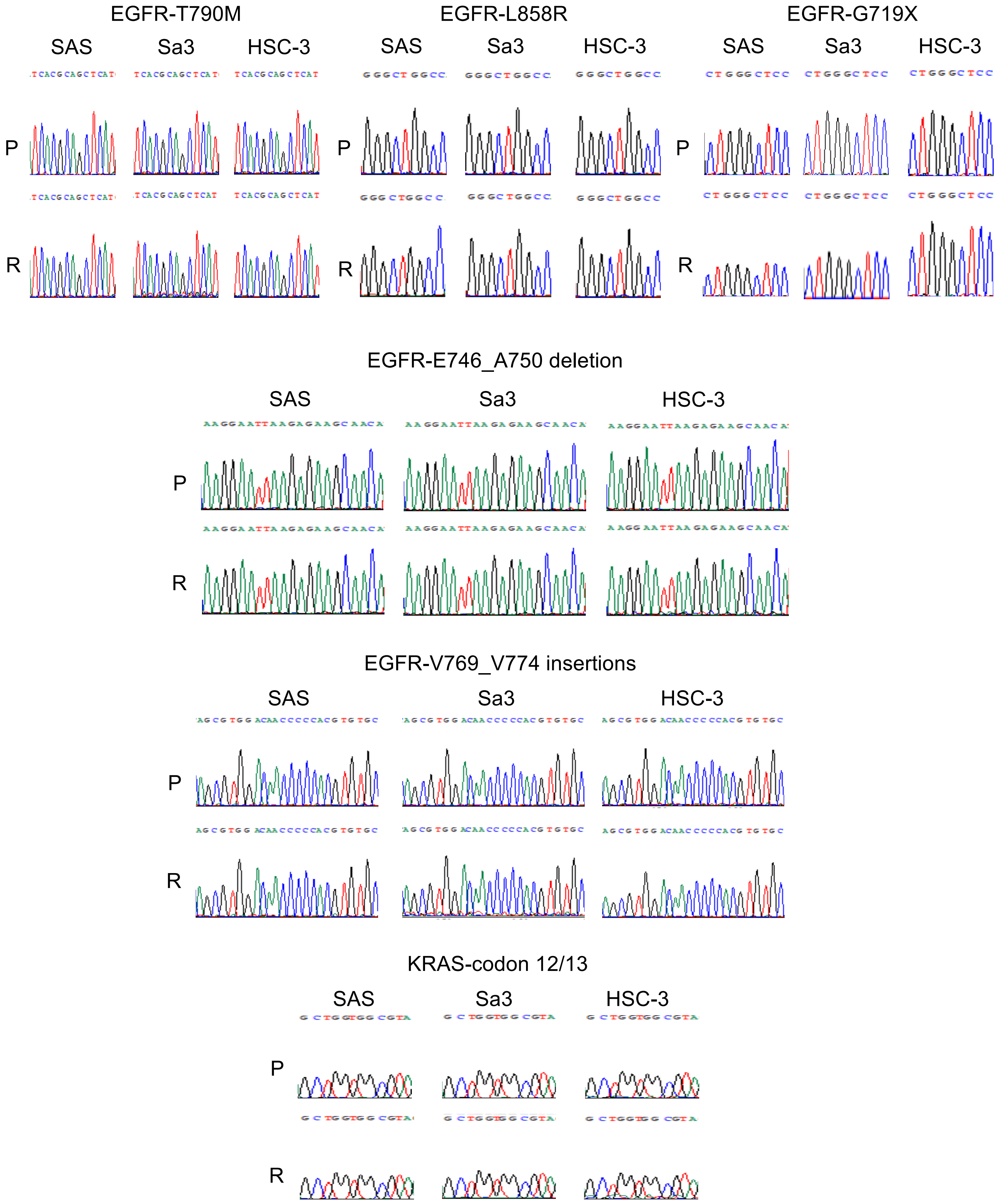

Supplementary Figure S2

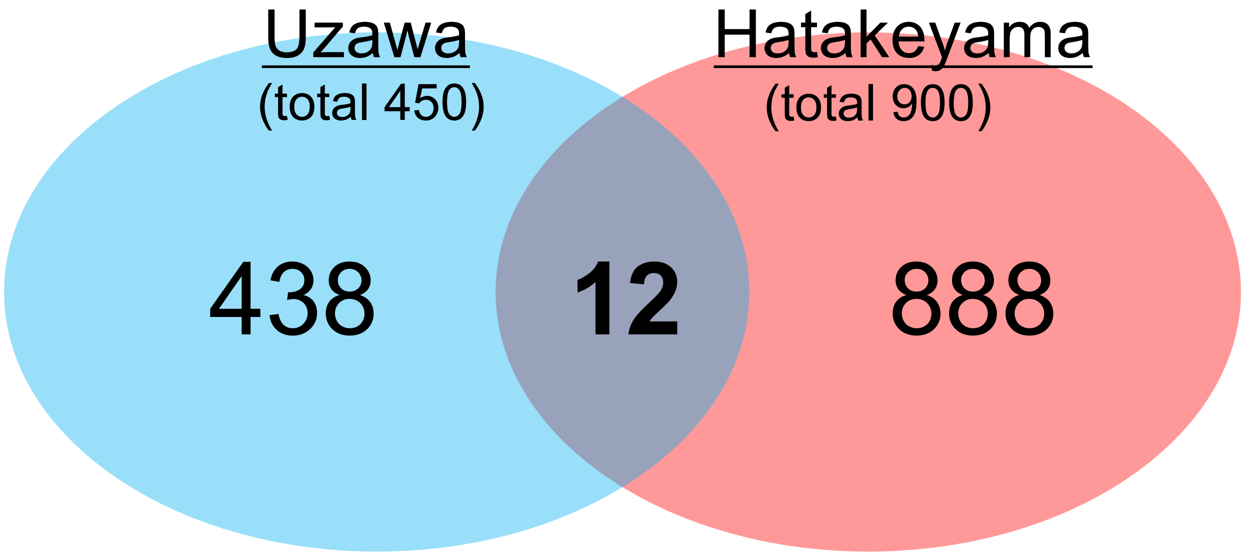

Supplementary Figure S3

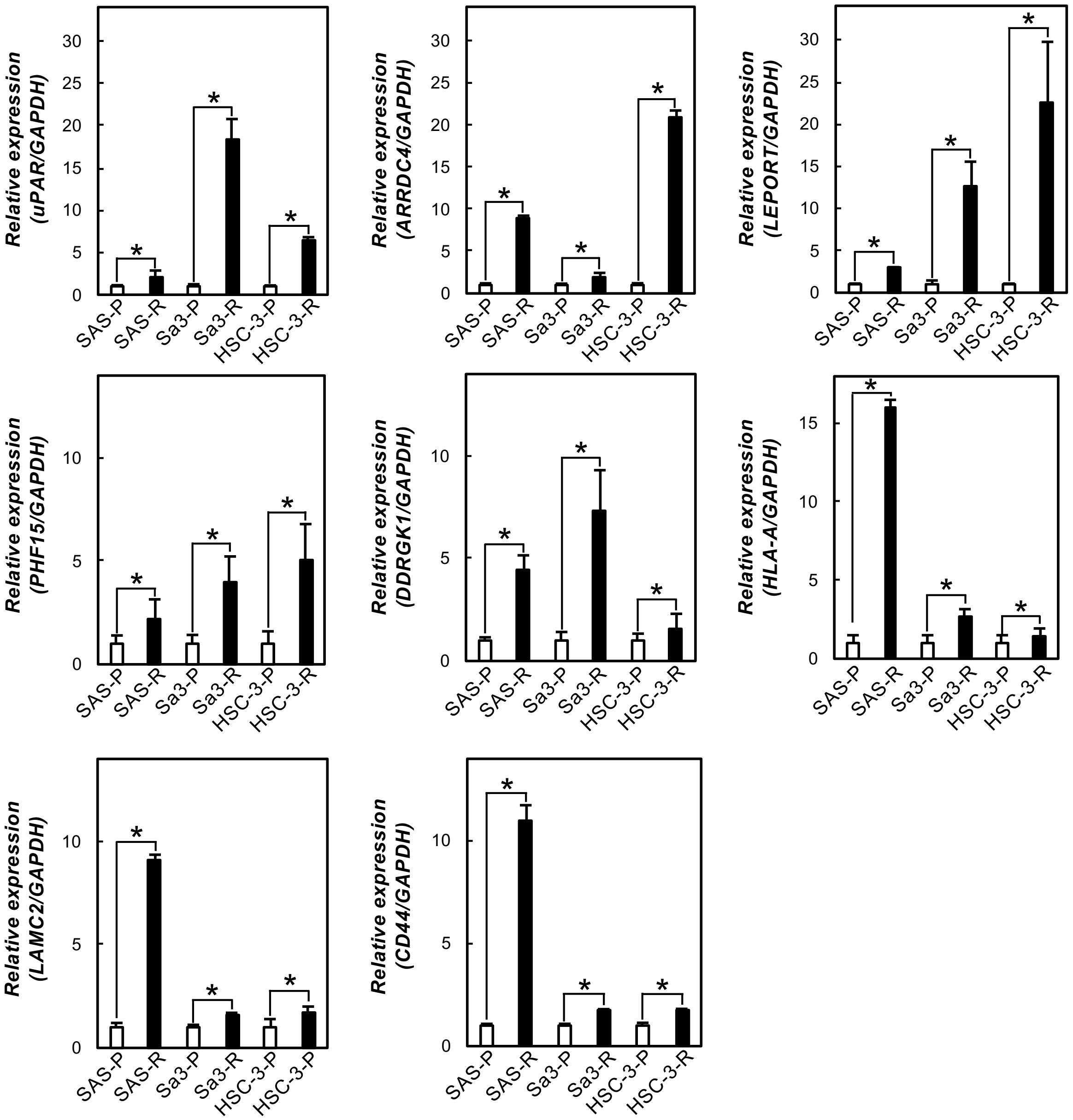

Supplementary Figure S4

(negative control of Figure 4B and Figure 5B)

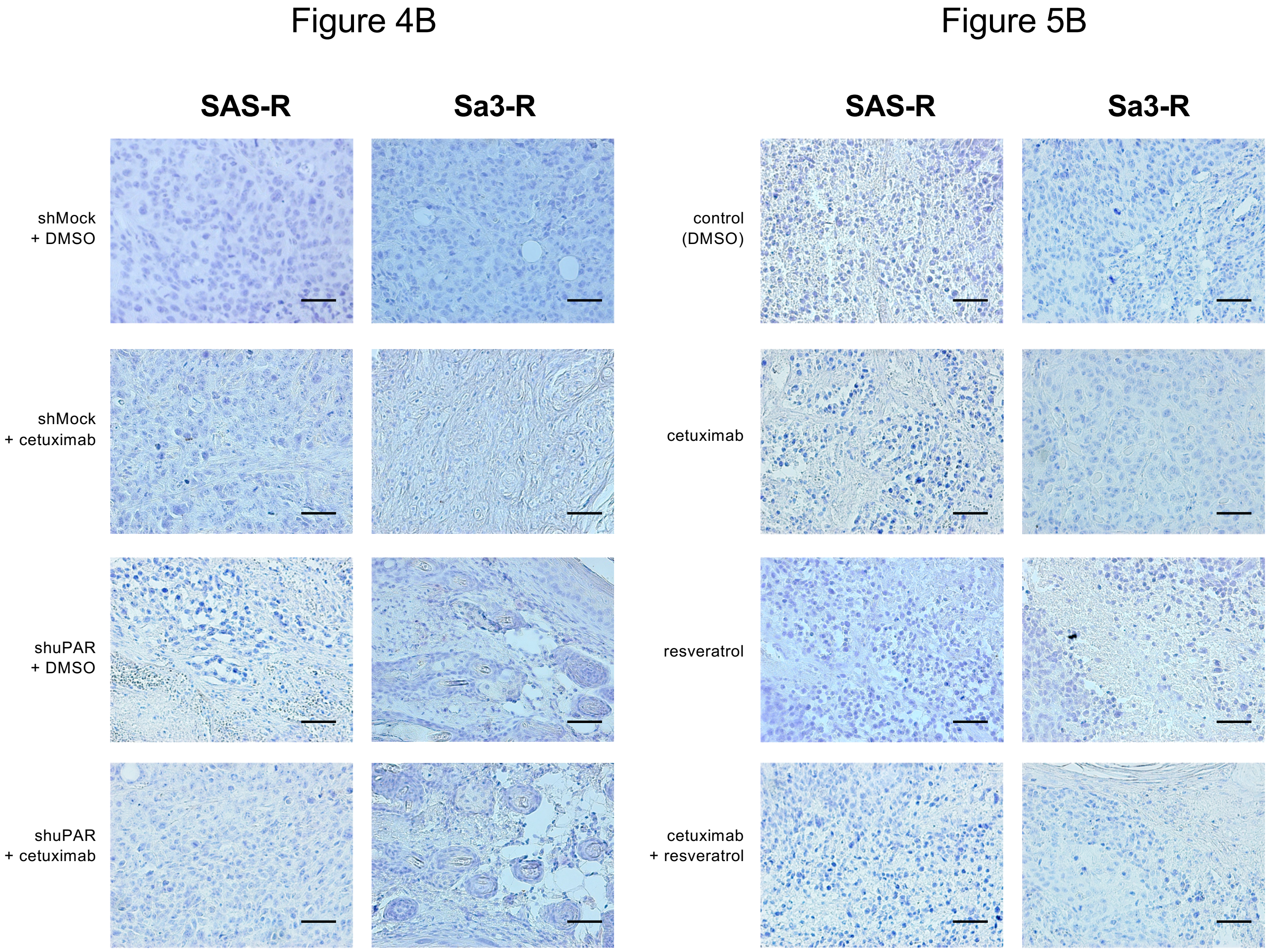

Supplementary Figure S5

A

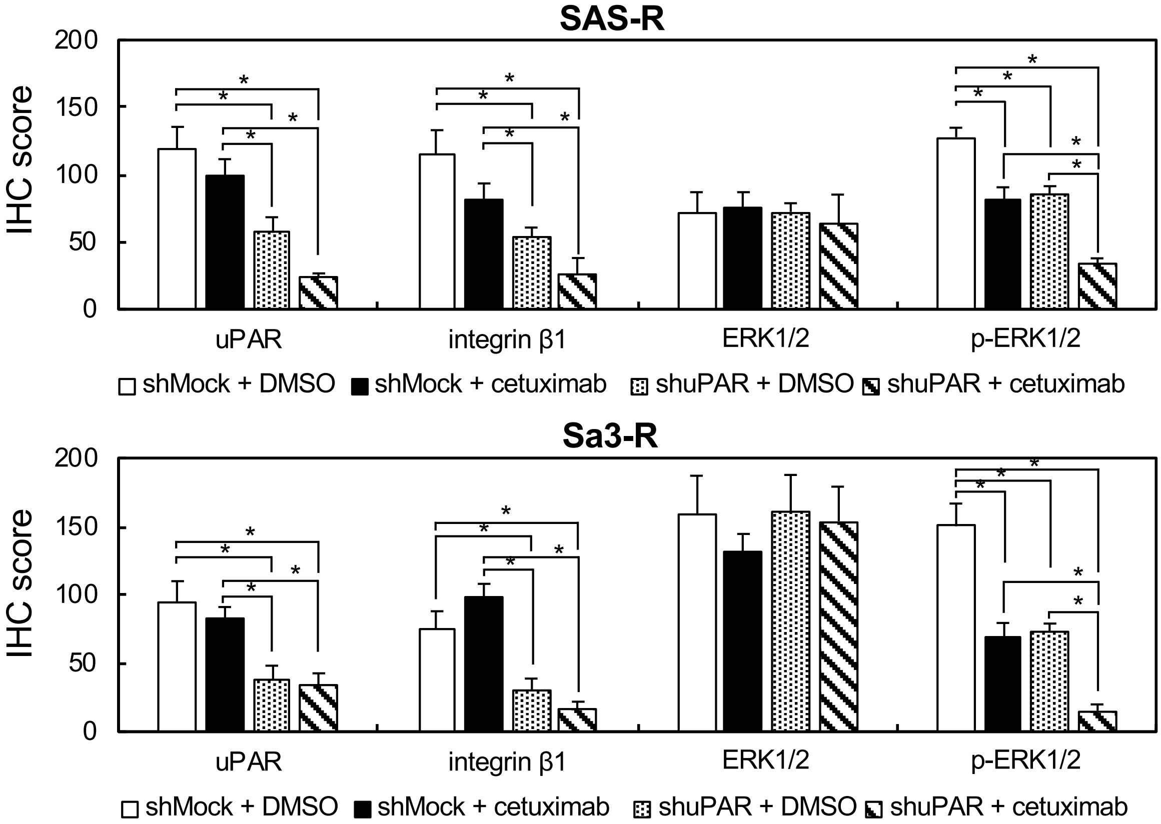

B

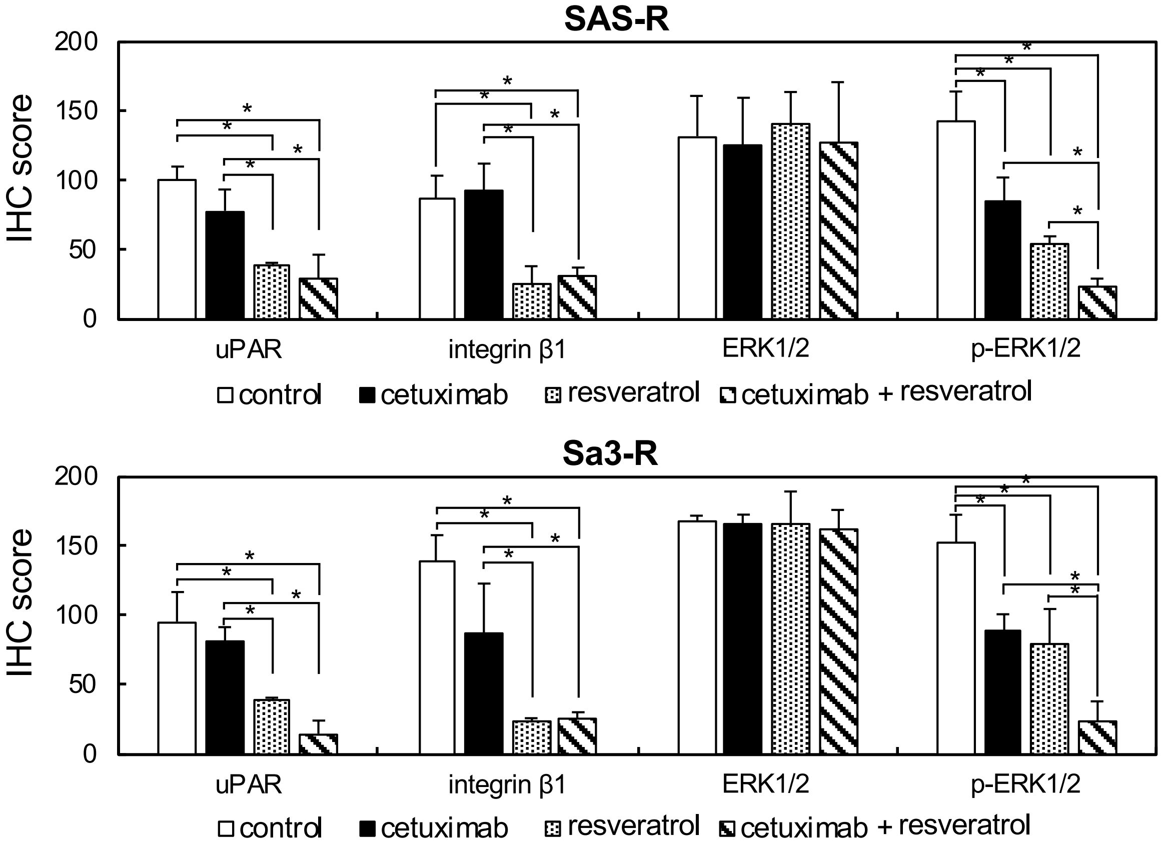

Supplementary Figure S6

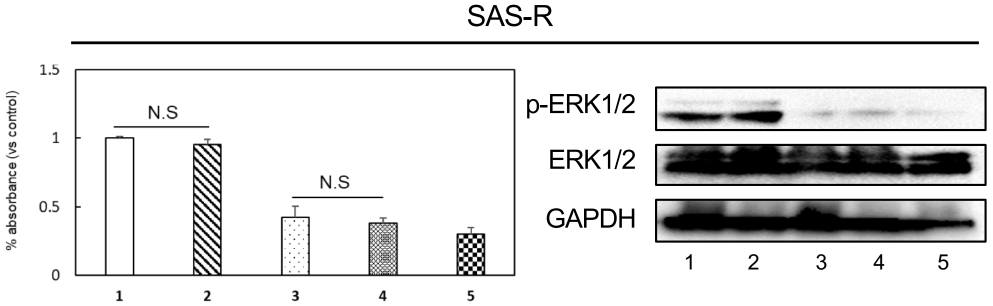

	1	2	3	4	5
**Cetuximab**	-	+	+	+	+
**Cenupatide**	-	-	+	+	-
**Resveratrol**	-	-	-	+	+

Supplementary Figure S7

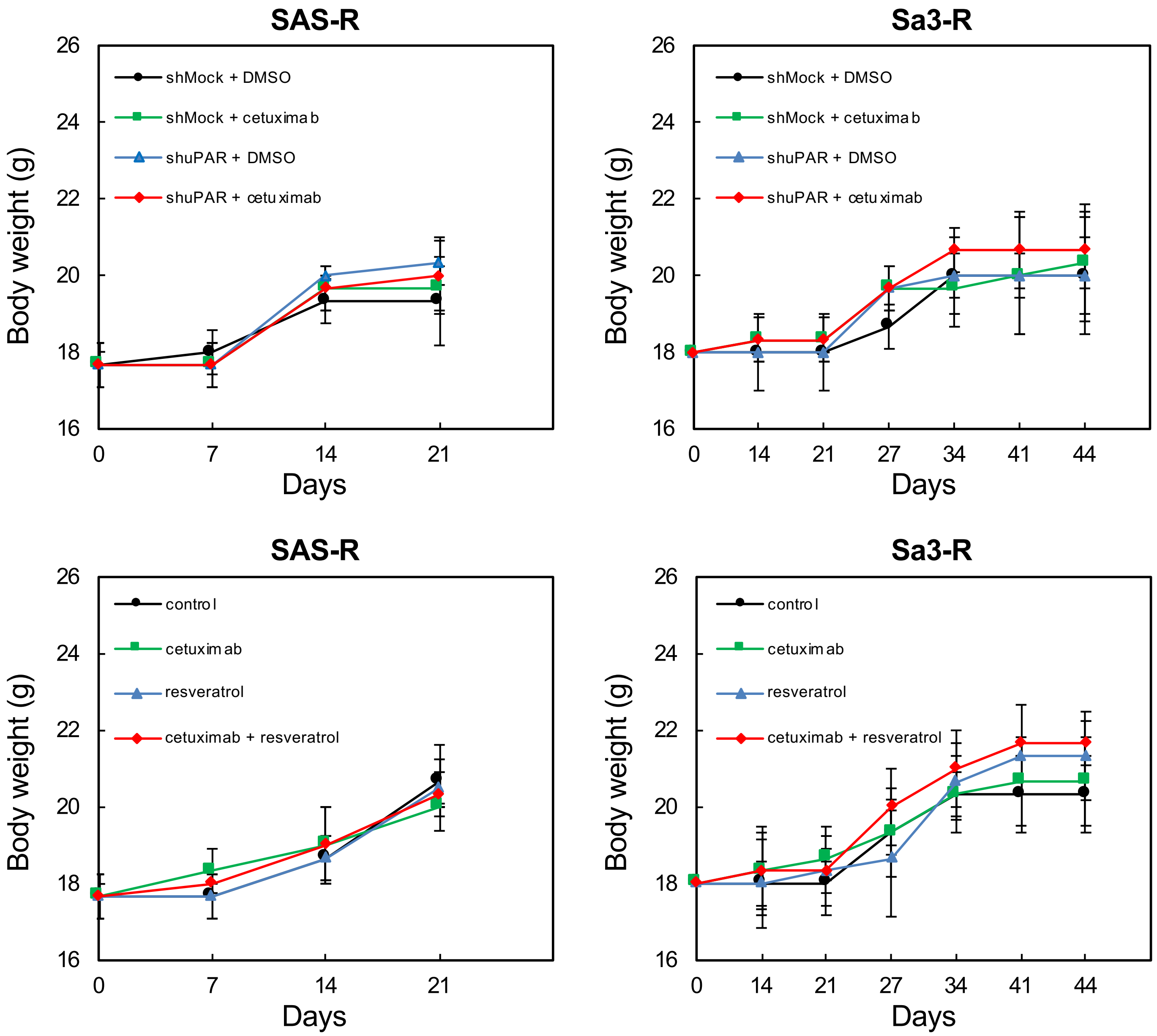

Supplementary Figure S8

A

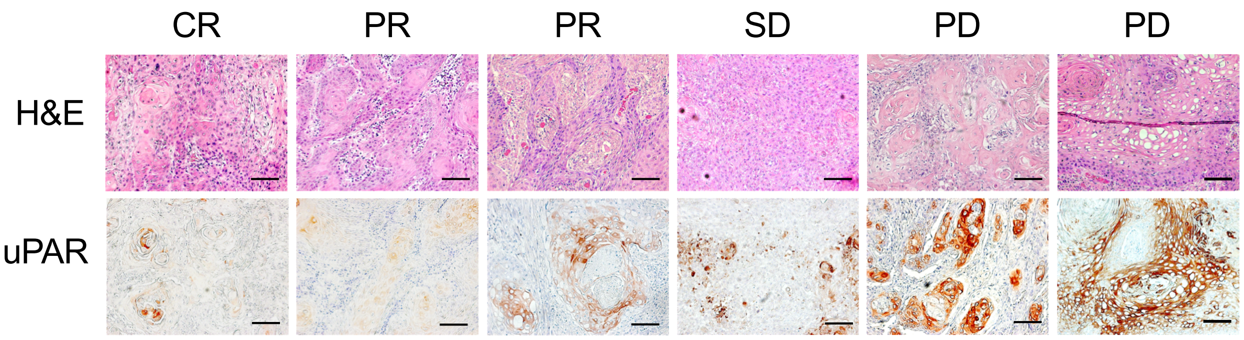

B

uPAR status	Primary OSCCs from cetuximab treated patients					
	CR	PR	PR	SD	PD	PD
high positive	0.9656	0.2269	3.1929	1.6129	6.1805	12.6392
positive	2.6515	1.5103	7.1658	3.4399	14.0731	16.8283
low positive	21.5223	30.681	20.475	25.0592	29.7322	31.1993
IHC score	29.7221	34.3823	44.3853	36.7777	76.4199	102.7735

Supplementary Figure S9

(Full length blots of Figure 1C)

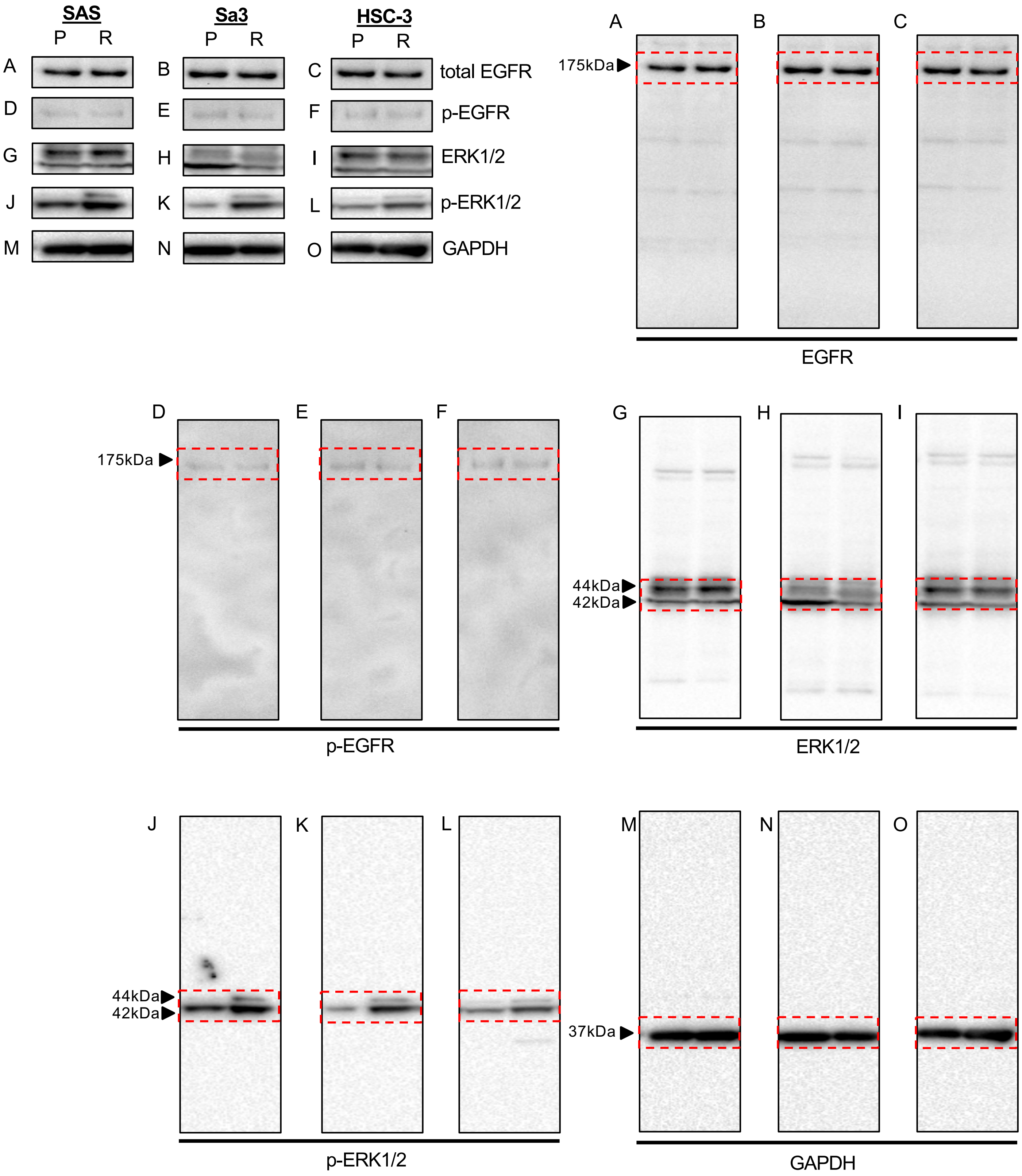

Supplementary Figure S10

(Full length blots of Figure 2A)

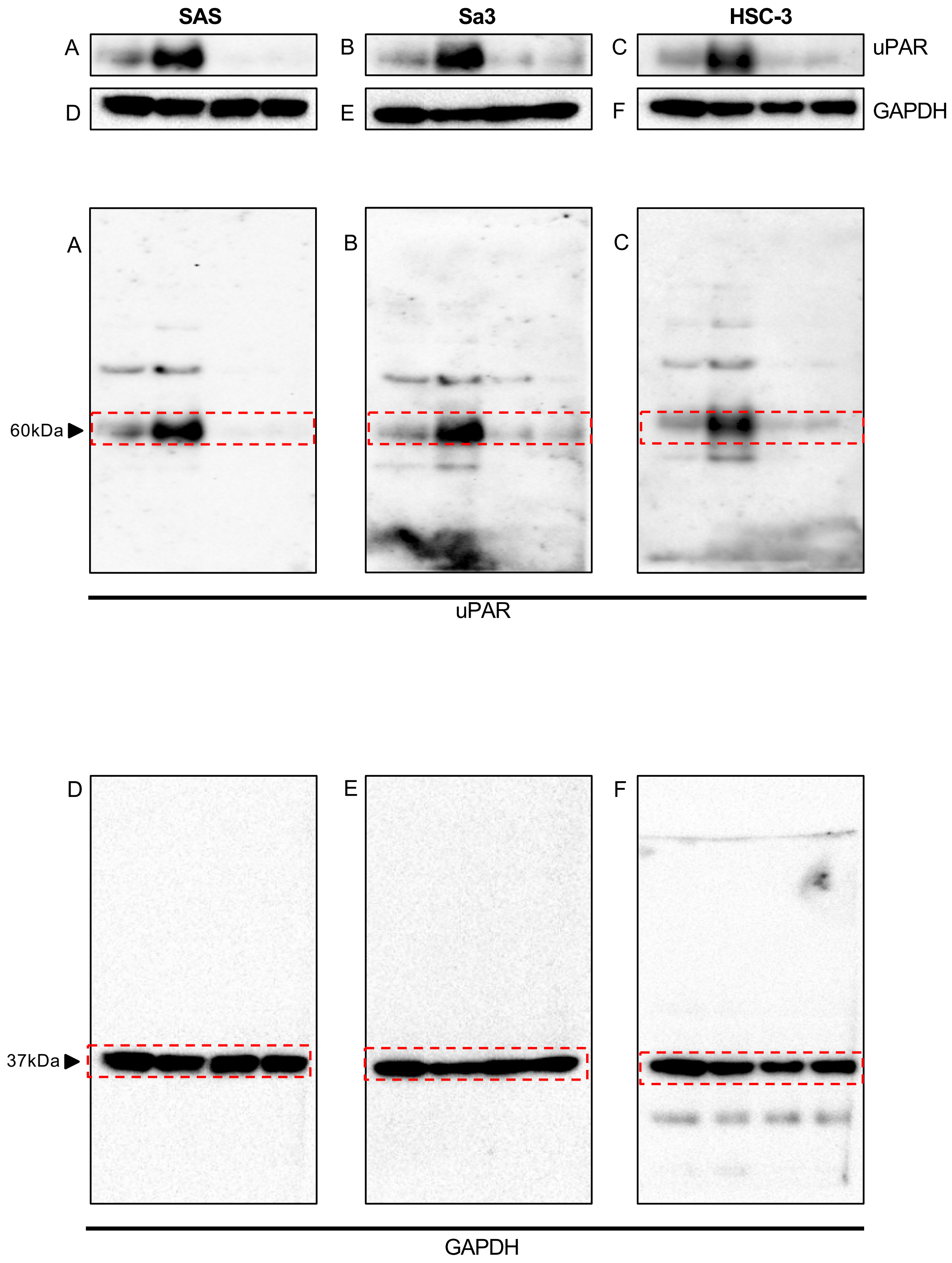

Supplementary Figure S11

(Full length blots of Figure 2B (SAS))

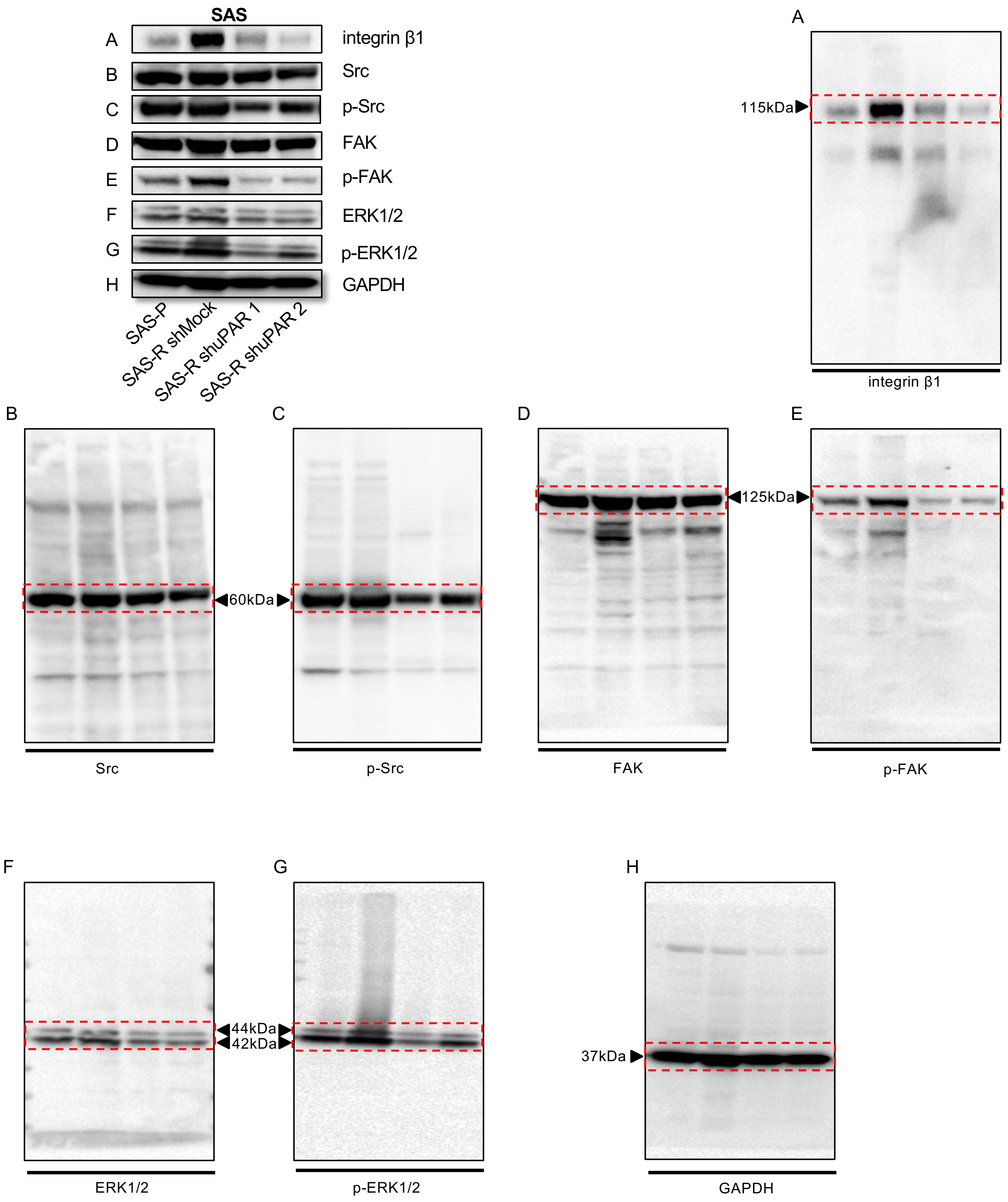

Supplementary Figure S12

(Full length blots of Figure 2B (Sa3))

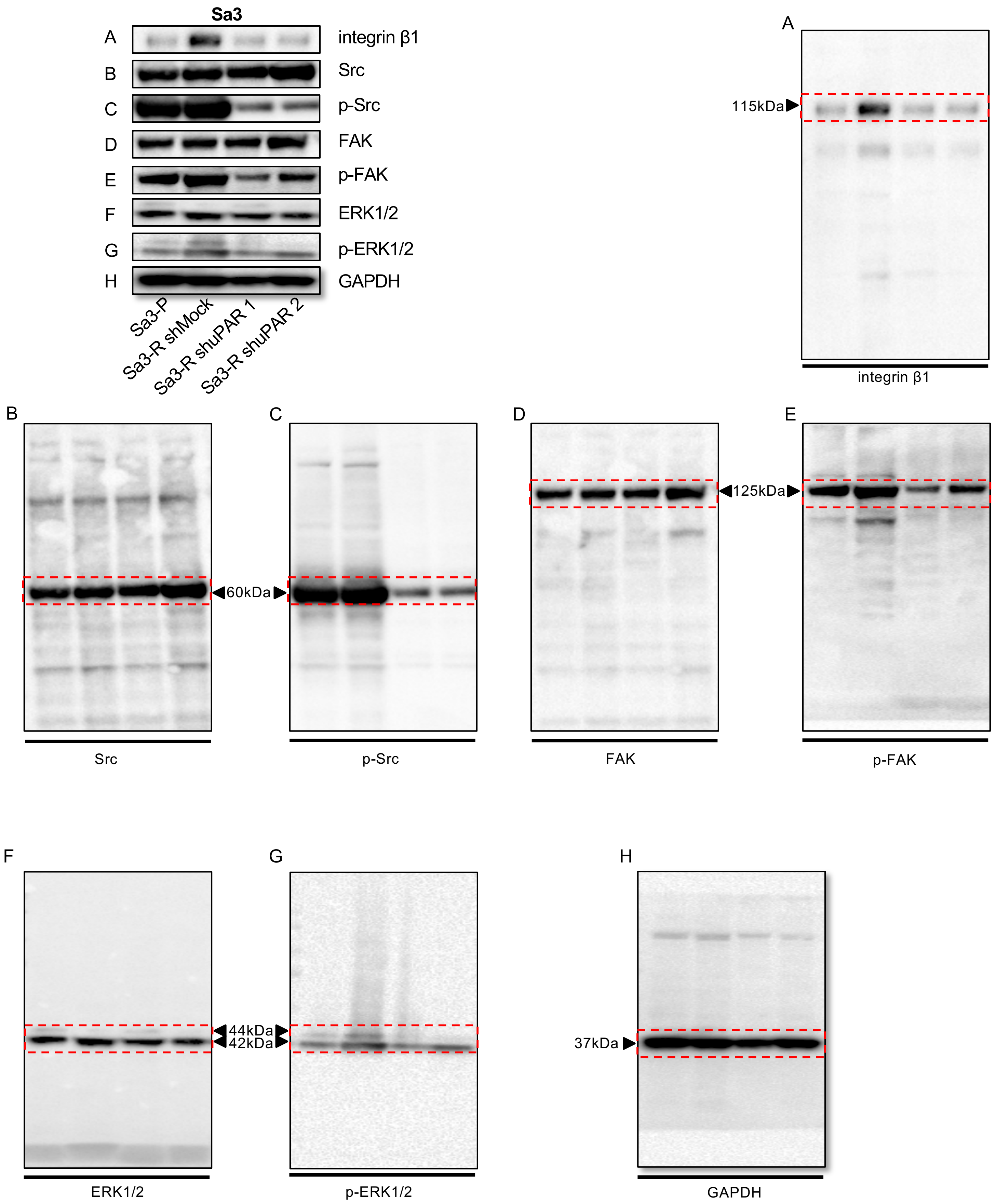

Supplementary Figure S13

(Full length blots of Figure 2B (HSC-3))

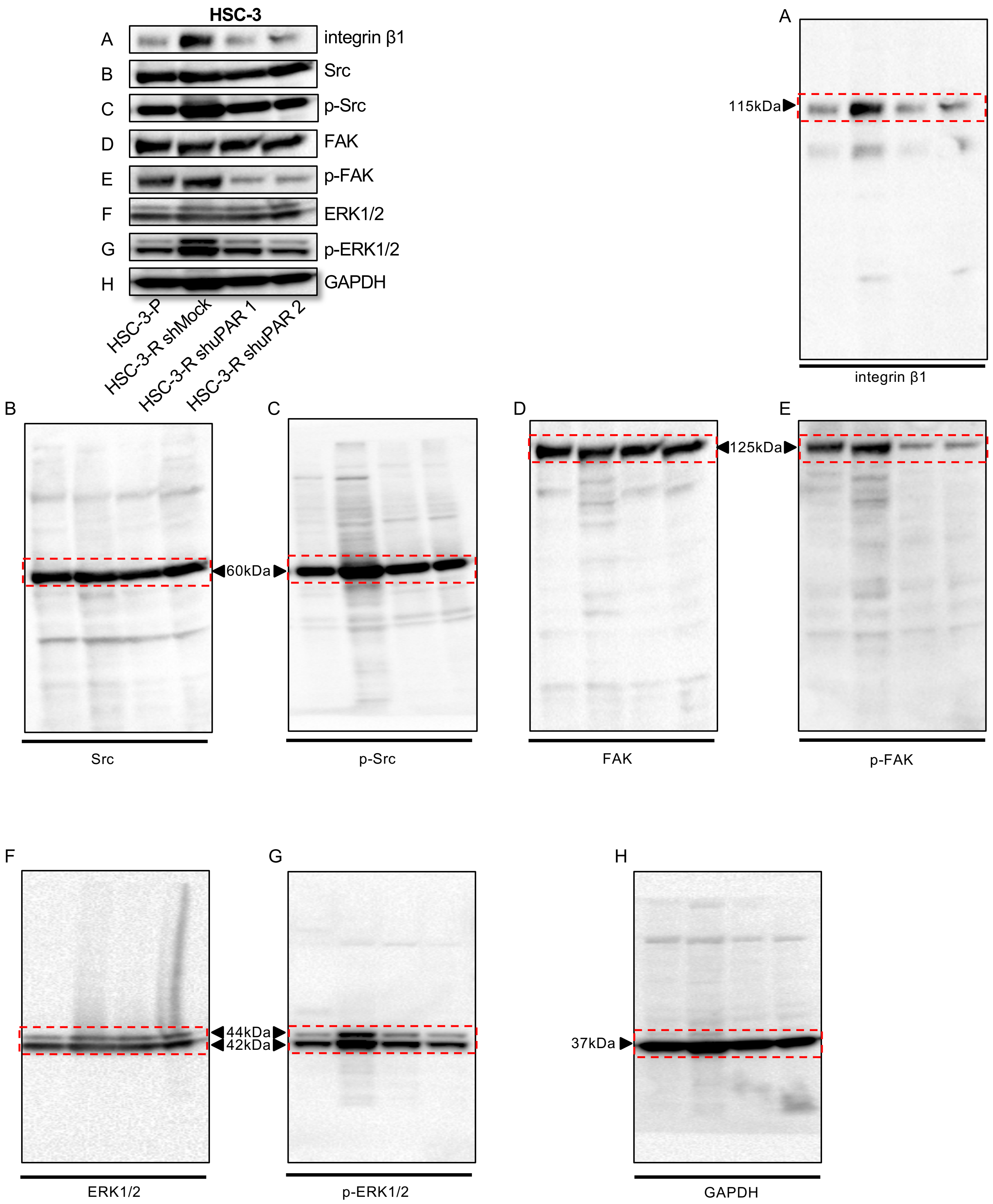

Supplementary Figure S14

(Full length blots of Figure 2C)

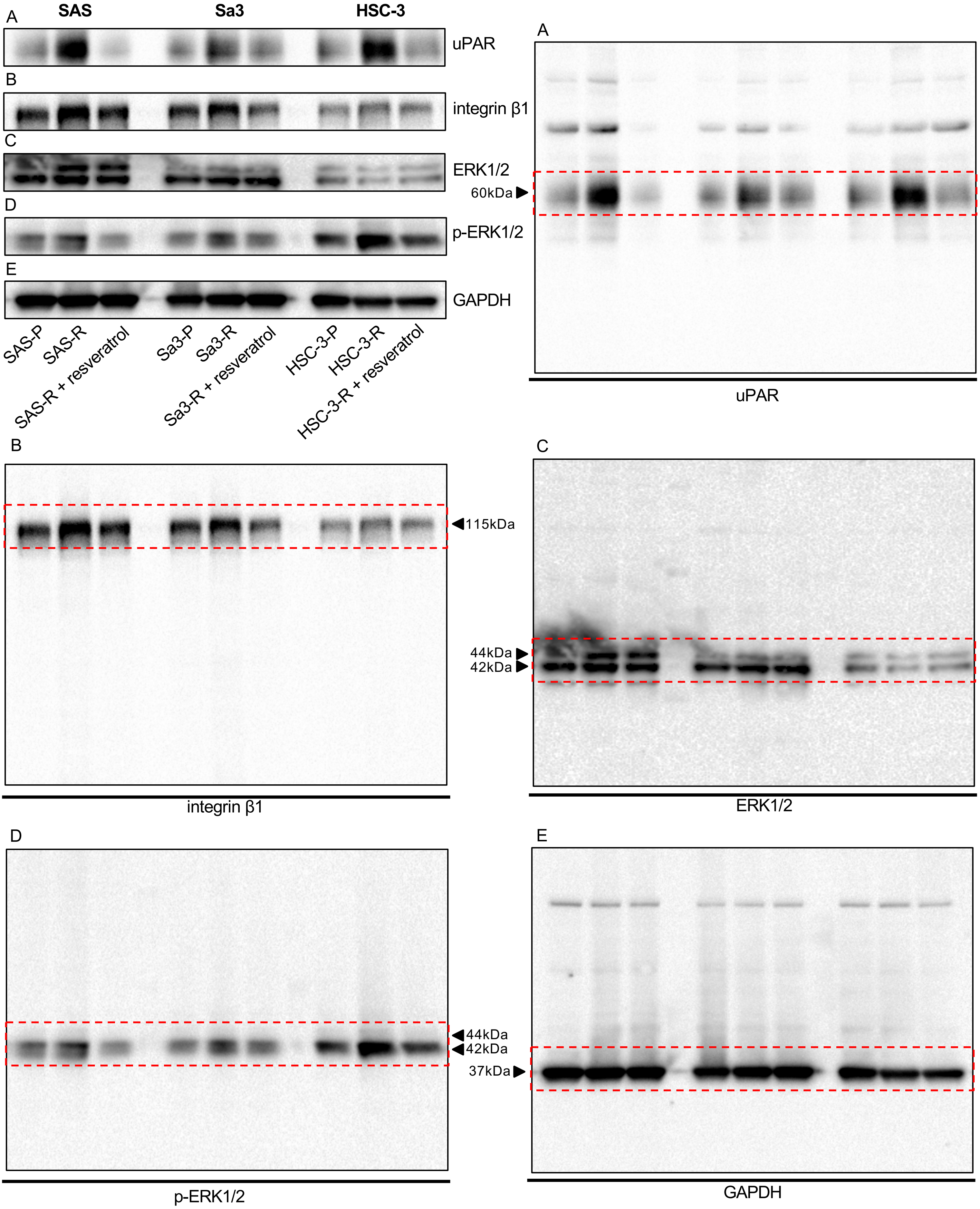

Supplementary Figure S15

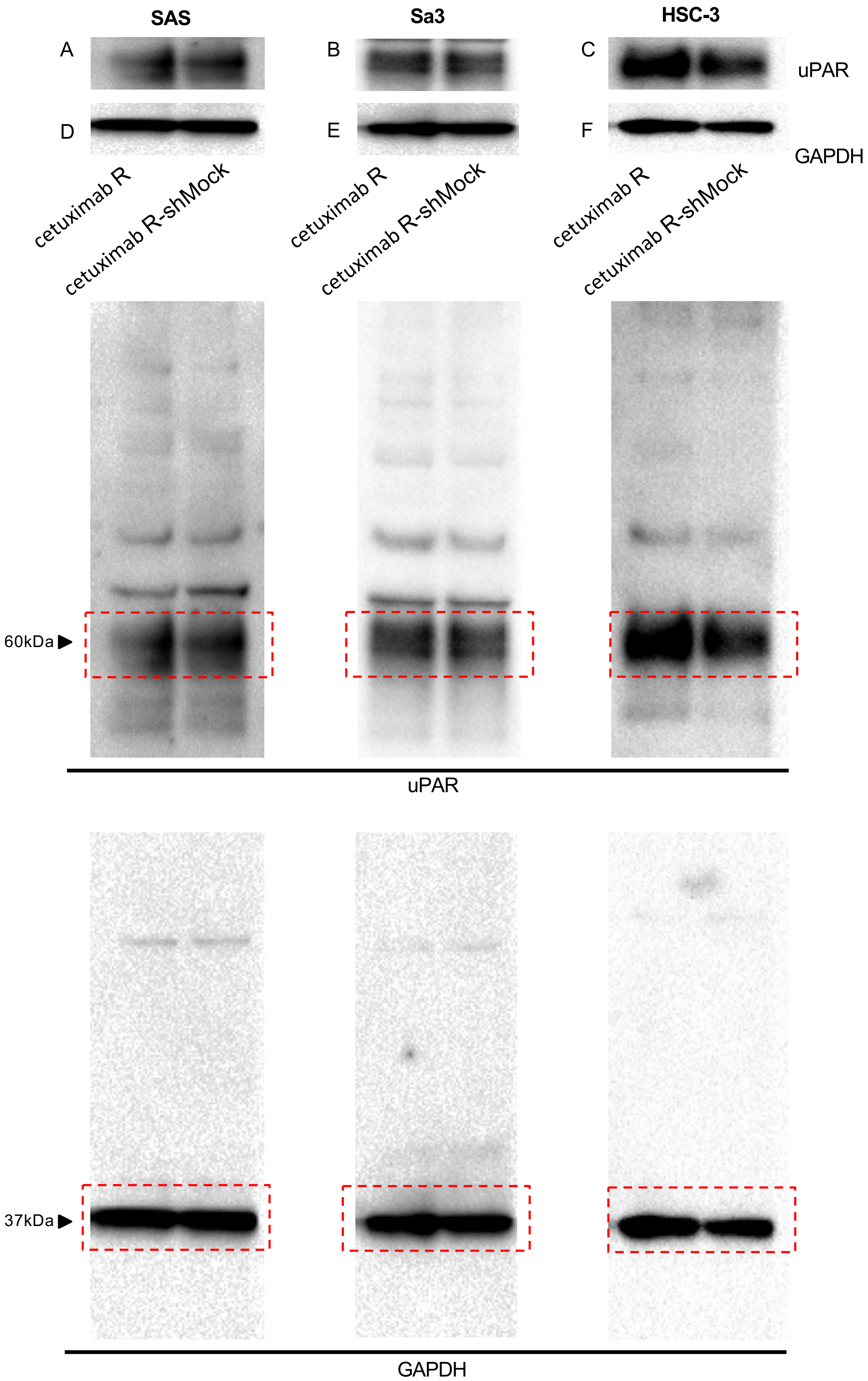

Supplementary Figure S16

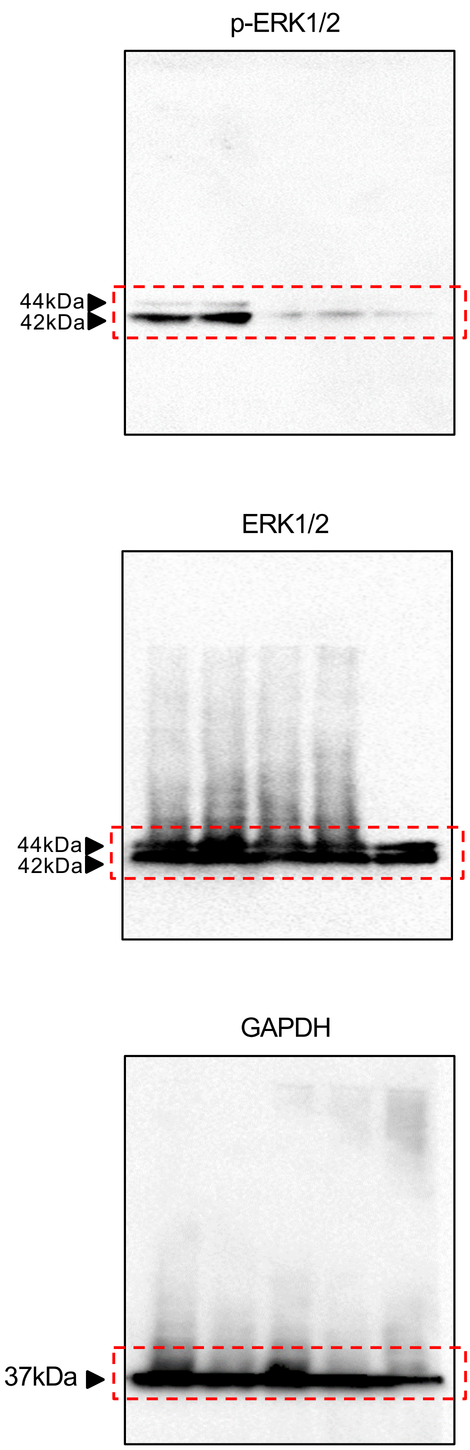

Supplement: Supplementary file 1 — Supplementary Information [file 41598_2019_48717_MOESM1_ESM.pdf]
