# Supplementary material for: Resveratrol Targets Urokinase-Type Plasminogen Activator Receptor Expression to Overcome Cetuximab-Resistance in Oral Squamous Cell Carcinoma
Source: Sci Rep. 2019 Aug 21;9:12179. doi: 10.1038/s41598-019-48717-w (PMC6704133; doi:10.1038/s41598-019-48717-w)
Supplement: Supplementary file 2 — Supplementary Table S1 [file 41598_2019_48717_MOESM2_ESM.pdf]

Supplementary information for

Resveratrol Targets Urokinase-Type Plasminogen Activator Receptor Expression to Overcome Cetuximab-Resistance in Oral Squamous Cell Carcinoma

Katsuhiro Uzawa[1,2]*, Antonio L Amelio[3,4,5]*, Atsushi Kasamatsu[2], Tomoaki Saito[2], Akihiro Kita[2], Megumi Fukamachi[1], Yuki Sawai[1], Yuriko Toeda[1], Keitaro Eizuka[1], Fumihiko Hayashi[1], Ikuko Kase-Kato[1], Masataka Sunohara[6], Manabu Iyoda[2], Kazuyuki Koike[2], Dai Nakashima[2], Katsunori Ogawara[1], Yosuke Sakamoto-Endo[2], Masashi Shiiba[7], Yuichi Takiguchi[7], Mitsuo Yamauchi[3] and Hideki Tanzawa[1,2]

[1] Department of Oral Science, Graduate School of Medicine, Chiba University, 1-8-1 Inohana, Chuo-ku, Chiba 260-8670, Japan
[2] Department of Dentistry and Oral-Maxillofacial Surgery, Chiba University Hospital, 1-8-1 Inohana, Chuo-ku, Chiba 260-8677, Japan
[3] Division of Oral and Craniofacial Health Sciences, University of North Carolina at Chapel Hill, Chapel Hill, NC 27599-7455, USA
[4] Lineberger Comprehensive Cancer Center, University of North Carolina at Chapel Hill, Chapel Hill, NC 27599-7455, USA
[5] Biomedical Research Imaging Center, University of North Carolina at Chapel Hill, Chapel Hill, NC 27599-7455, USA
[6] Department of Anatomy, School of Life Dentistry at Tokyo, Nippon Dental University, 1-9-20 Fujimi, Chiyoda-ku, Tokyo 102-8159, Japan
[7] Department of Medical Oncology, Graduate School of Medicine, Chiba University, 1-8-1 Inohana, Chuo-ku, Chiba 260-8670, Japan

***Corresponding Authors:**
Katsuhiro Uzawa, DDS, PhD, FIBCSOMS
Editorial Board Member of Scientific Reports (Cancer Category)
Department of Oral Science
Graduate School of Medicine, Chiba University
1-8-1 Inohana, Chuo-ku
Chiba, 260-8670, Japan
Phone: +81-43-226-2300
E-mail: uzawak@faculty.chiba-u.jp

Antonio L. Amelio, PhD
Division of Oral and Craniofacial Health Sciences
Lineberger Comprehensive Cancer Center
University of North Carolina at Chapel Hill
Chapel Hill, NC 27599-7455, USA
Phone: (919) 537-3309
E-mail: alamelio@email.unc.edu

Supplementary Table S1. Candidate genes increased significantly in the cetuximab-resistant cells

Probe ID	Symbol	Name	parental cells			resistant		
			SAS	Sa3	HSC-3	SAS	Sa3	HSC-3
A_23_P97700	TXNIP	thioredoxin interacting protein	1072.84	96.1433	122.343	39547.1	26784.9	43156.9
A_21_P0014894			19.2041	5.05957	3.52397	383.592	506.954	562.833
A_24_P178065	PHLDB2	pleckstrin homology like domain family B member 2	13.8285	20.1503	3.63305	111.05	906.309	526.248
A_23_P338479	CD274	CD274 molecule	26.1045	62.3147	7.77029	132.007	1381.61	1095.08
A_33_P3346936	MON2	MON2 homolog, regulator of endosome-to-Golgi trafficking	20.075	10.5618	6.62888	181.78	192.217	571.45
A_21_P0000073	OSMR	oncostatin M receptor	310.784	578.109	153.537	5515.16	7922.06	8043.04
A_33_P3237567	HLA-A	major histocompatibility complex, class I, A	91.2334	43.6988	78.5075	955.11	1570.65	2430.57
A_33_P3343516	LOC100129617	uncharacterized LOC100129617	21.0469	21.1759	6.75365	214.068	1209.58	121.395
A_33_P3408918	SAA2	serum amyloid A2	396.513	279.23	130.226	12841.1	1237.43	6225.57
A_21_P0000152	CD44	CD44 molecule (Indian blood group)	38.493	91.7745	29.5994	461.681	1615.37	959.117
A_33_P3424462	CNST	consortin, connexin sorting protein	19.4103	23.4129	6.99491	171.051	309.985	330.296
A_33_P3390024	EXOC7	exocyst complex component 7	47.6448	53.0321	36.1884	323.572	1931.16	763.842
A_21_P0009009			4.00594	7.24498	5.20862	77.1598	71.3591	142.201
A_33_P3310286			7.96423	30.2674	3.56487	70.978	320.991	169.763
A_33_P3299781	DDRGK1	DDRGK domain containing 1	246.762	141.185	138	2307.39	3994.99	2184.17
A_33_P3230528	MPRIP	myosin phosphatase Rho interacting protein	68.8041	216.534	62.2419	976.346	3601.35	1071.37
A_24_P332647	SSH1	slingshot protein phosphatase 1	45.4705	84.7454	14.6712	333.445	854.304	800.009
A_33_P3240158			8.30575	9.33147	9.88333	38.994	246.562	304.263
A_21_P0010978			27.4653	16.4641	40.8952	163.055	527.278	717.989
A_33_P3331080	SEC24A	SEC24 homolog A, COPII coat complex component	32.5051	26.3027	15.9556	174.173	683.952	372.689
A_33_P3263538			291.852	202.66	442.86	3114.85	3581.39	7429.44
A_33_P3239569	RPS21	ribosomal protein S21	74.2662	98.0833	101.929	345.916	4623.62	1449.85
A_33_P3346937	MON2	MON2 homolog, regulator of endosome-to-Golgi trafficking	34.3129	19.2826	32.8026	246.709	314.873	842.787
A_33_P3379571	MAP3K9	mitogen-activated protein kinase kinase kinase 9	94.6864	101.449	44.9893	859.577	2736.64	485.738
A_33_P3405500	SACS	sacsin molecular chaperone	40.5012	58.5556	22.3989	280.553	560.654	869.931
A_33_P3240757	IGSF3	immunoglobulin superfamily member 3	25.0734	10.8184	29.262	315.74	260.053	237.006
A_23_P81898	UBD	ubiquitin D	19.632	26.2398	3.48179	212.879	123.76	155.187
A_21_P0000535	LINC00173	long intergenic non-protein coding RNA 173	24.8036	5.54708	7.9904	72.6794	149.206	203.533
A_33_P3242952	FAM72A	family with sequence similarity 72 member A	138.951	369.307	37.9733	1131.17	1344.26	2544.24
A_23_P62642	CFAP45	cilia and flagella associated protein 45	23.6777	18.1338	3.39429	237.379	197.03	61.4274
A_33_P3390713			25.3995	3.88746	7.05761	214.986	70.8255	87.159
A_21_P0014428	LOC101927372	uncharacterized LOC101927372	211.603	37.9837	206.457	1600.82	1286.88	1399.83
A_24_P230938	MORN4	MORN repeat containing 4	57.1549	78.5486	155.563	484.507	1454.4	1684.29
A_23_P426305	AOC3	amine oxidase copper containing 3	21.0543	9.4982	7.30544	102.7	159.966	141.441
A_24_P34155	RUNX1	runt related transcription factor 1	73.872	70.1697	33.5063	481.936	983.947	570.998
A_24_P376129	DFNB31	deafness, autosomal recessive 31	17.0335	7.08285	15.3769	124.219	208.067	109.729
A_21_P0006372	LOC101929014	uncharacterized LOC101929014	54.6361	23.4643	18.1943	525.005	81.5855	745.272
A_33_P3420862	PAGE2B	PAGE family member 2B	67.1685	7.50783	6.67832	196.46	153.025	144.11
A_33_P3336925	PDLIM5	PDZ and LIM domain 5	212.494	309.649	131.111	1710.27	1639.86	3934.27
A_23_P339818	ARRDC4	arrestin domain containing 4	103.384	31.3877	78.0175	862.509	317.649	1098.88
A_21_P0000086	GOLGA3	golgin A3	20.2615	10.3761	8.56644	103.103	52.1376	389.874
A_23_P424561	RHOV	ras homolog family member V	79.1197	99.5614	82.9286	393.526	2369.68	747.345
A_21_P0001979			4.56622	11.7135	9.09323	181.005	64.5801	44.3362
A_33_P3280597			22.1495	23.2356	24.3925	257.716	195.917	260.008
A_33_P3363720	ZNF436-AS1	ZNF436 antisense RNA 1	32.5272	45.7925	77.4278	181.929	701.561	940.649
A_23_P53370	RND1	Rho family GTPase 1	15.0668	14.5192	15.4502	55.3304	268.216	235.184
A_19_P00321743	LOC100130938	uncharacterized LOC100130938	171.85	42.7271	40.1433	546.954	1315.99	422.327
A_23_P111132	HSPA1A	heat shock protein family A (Hsp70) member 1A	4887.28	2699.81	3751.2	15756.3	70713.3	42808.7
A_33_P3228642			37.0459	30.6396	20.4634	183.17	831.461	138.695
A_19_P00805006	MALAT1	metastasis associated lung adenocarcinoma transcript 1	708.915	188.72	269.591	1615.99	3813	5186.61
A_33_P3339361	ARHGAP11A	Rho GTPase activating protein 11A	21.0774	21.6628	6.69812	76.4086	142.457	246.137
A_23_P169017	DEFB103B	defensin beta 103B	27.694	22.3019	6.24857	211.91	50.131	315.151
A_24_P226278	JADE2	jade family PHD finger 2	121.198	394.317	286.461	985.836	4069.28	2882.26
A_32_P70927	PAGE2	PAGE family member 2	32.0754	4.06481	3.51969	82.4085	72.3944	60.6788
A_24_P130936	DDX3Y	DEAD-box helicase 3 Y-linked	36.9722	25.3327	23.7872	359.785	193.258	250.39
A_23_P144807	8-Sep	septin 8	7.90781	4.94759	4.39266	46.6296	49.3815	58.2356
A_33_P3330236			27.575	27.8482	17.9417	244.665	443.08	98.9697
A_23_P103110	MAFF	MAF bZIP transcription factor F	280.724	331.975	118.725	1017.3	4868.66	1701.98
A_23_P374902	CLDND2	claudin domain containing 2	7.12309	14.9486	22.0703	44.285	333.445	120.764
A_21_P0012948			8.95472	10.2219	4.10119	22.8988	119.552	102.415
A_24_P45446	GBP4	guanylate binding protein 4	102.825	78.3971	35.9632	852.591	183.762	1357.83
A_21_P0000140	LEPROT	leptin receptor overlapping transcript	589.763	685.108	487.175	5742.92	3688.93	6683.25
A_23_P118392	RASD1	ras related dexamethasone induced 1	40.0419	27.3902	26.7167	121.272	562.359	301.867
A_33_P3328637	PRRG1	proline rich and Gla domain 1	60.9817	53.6597	31.4689	468.549	240.003	627.598
A_23_P16469	PLAUR	plasminogen activator, urokinase receptor	523.403	403.15	140.036	3748.74	2708.49	1975.11
A_33_P3217020	RAPH1	Ras association (RalGDS/AF-6) and pleckstrin homology domains 1	72.8376	92.8604	101.395	222.613	2413.39	857.735
A_21_P0000295	SNORA67	small nucleolar RNA, H/ACA box 67	539.334	1579.7	177.928	4642.72	7638.99	2784.75
A_19_P00315452	LOC100130938	uncharacterized LOC100130938	214.874	52.808	59.5999	670.15	1009.44	621.961
A_33_P3415430	HSPA1B	heat shock protein family A (Hsp70) member 1B	2172.87	1750.22	4993.98	6966.14	52383.4	31948.7
A_21_P0008727			3.83387	10.4382	7.34173	74.0263	24.4419	99.4205
A_32_P196142	LOC100130938	uncharacterized LOC100130938	247.071	59.9784	73.4282	823.368	1184.06	663.146
A_21_P0014789			3.49901	3.49105	3.54478	13.6886	114.95	15.604
A_23_P29769	WWTR1	WW domain containing transcription regulator 1	104.714	116.261	33.8843	464.917	847.633	589.763
A_24_P305784	SPANXB1	SPANX family member B1	5.57476	7.35558	8.07849	26.9905	60.5201	113.053
A_33_P3259821	DOCK9	dedicator of cytokinesis 9	111.573	76.3372	59.5054	722.188	1008	385.69
A_24_P382187	IGFBP4	insulin like growth factor binding protein 4	86.4201	72.1176	17.0535	264.918	651.028	338.741
A_21_P0001258			4.79358	4.22635	5.29449	14.3199	39.735	102.961
A_33_P3299309	RPL28	ribosomal protein L28	5.3286	13.0684	11.6951	35.0061	118.769	105.872
A_33_P3246505	MAP3K8	mitogen-activated protein kinase kinase kinase 8	72.7418	103.224	61.6982	345.604	865.978	818.67
A_33_P3353343	SRRM2	serine/arginine repetitive matrix 2	44.8205	86.4507	67.0975	241.259	1536.76	367.272
A_33_P3272553	NCAPH2	non-SMC condensin II complex subunit H2	63.8379	133.954	87.0939	352.941	1596.81	687.919
A_33_P3284763	DMD	dystrophin	15.5613	35.3845	22.9918	54.7402	424.857	277.557
A_21_P0004591	MLLT4-AS1	AFDN divergent transcript	13.8257	14.7419	7.92102	88.8536	134.343	68.8041
A_21_P0005325			13.6875	8.32635	6.81518	24.33	140.235	110.407
A_24_P290770	OGDH	oxoglutarate dehydrogenase	48.3001	52.7584	49.1374	247.424	408.516	592.907
A_19_P00318213	HCG18	HLA complex group 18	112.115	83.1209	89.8889	264.968	1205.88	1214.68
A_33_P3381305			271.555	326.77	217.35	1606.72	1505.65	3672.52
A_33_P3311551	WLS	Wnt ligand secretion mediator	65.7306	101.314	55.3646	420.916	566.777	701.906
A_23_P160968	LAMC2	laminin subunit gamma 2	1768.24	907.735	985.443	7202.48	7273.53	13498.3
A_23_P154217	ITGB6	integrin subunit beta 6	37.4855	12.141	10.7132	248.576	64.9046	132.896
A_24_P152404	C10orf76	armadillo-like helical domain containing 3	215.264	151.071	143.971	964.054	1859.59	1139.17
A_33_P3237874	TROAP	trophinin associated protein	151.941	542.697	94.4219	824.49	2409.65	1676.85
A_21_P0000023	RIOK2	RIO kinase 2	13.0446	22.3789	11.8017	41.6884	159.421	216.265
A_23_P218131	INF2	inverted formin, FH2 and WH2 domain containing	326.073	782.245	386.85	2011.22	8132.61	2485.12
A_24_P250227	NR1D1	nuclear receptor subfamily 1 group D member 1	44.096	42.6855	139.066	226.837	755.156	629.427
A_24_P169688	MICB	MHC class I polypeptide-related sequence B	71.1282	130.286	46.2337	239.245	869.494	831.461

A_33_P3251932	BCL2L11	BCL2 like 11	152.152	156.729	133.262	532.344	2395.02	990.476
A_24_P50908	TRIM11	tripartite motif containing 11	118.147	396.183	101.852	620.269	2248.83	1337.87
A_33_P3225937	CRYZL1	crystallin zeta like 1	43.1672	58.4616	38.909	177.631	306.116	691.425
A_24_P123408	ABLIM3	actin binding LIM protein family member 3	38.683	107.007	59.6228	321.203	468.223	614.782
A_33_P3266719	TET2	tet methylcytosine dioxygenase 2	22.161	11.7392	19.4152	178.923	105.842	96.3697
A_33_P3230541			7.81274	7.612	3.72039	37.5506	81.4057	26.0705
A_33_P3213772	SRGAP2	SLIT-ROBO Rho GTPase activating protein 2	964.625	1202.39	537.734	10543	3709.94	5742.92
A_33_P3319261	EXD3	exonuclease 3'-5' domain containing 3	310.258	225.039	365.728	1150.06	3785.54	2100.21
A_33_P3325978			28.1263	79.3194	46.3107	159.58	394.944	571.731
A_23_P309381	HIST2H2AA4	histone cluster 2 H2A family member a4	852.257	174.573	964.054	3200.5	2664.11	5817.18
A_23_P201939	PPM1J	protein phosphatase, Mg2+/Mn2+ dependent 1J	344.718	127.364	264.17	1971.02	1262.75	1610.5
A_33_P3280603	PPP4R1L	protein phosphatase 4 regulatory subunit 1 like (pseudogene)	21.7913	19.0408	11.2273	141.311	70.1322	162.443
A_33_P3236392	PVRL4	nectin cell adhesion molecule 4	481.254	326.996	348.984	1980.69	6469.8	1423.98
A_23_P258410	WNT7A	Wnt family member 7A	97.3811	91.9476	138.612	317.911	784.785	1614.16
A_33_P3320888	CREB3L2	cAMP responsive element binding protein 3 like 2	46.1074	30.4152	32.7031	167.705	309.152	286.272
A_24_P937325	FGD4	FYVE, RhoGEF and PH domain containing 4	27.3234	18.148	20.5572	127.403	141.801	180.661
A_19_P00806098	LOC100130938	uncharacterized LOC100130938	181.26	70.149	66.6124	620.303	872.661	499.633
A_33_P3327097	MTMR1	myotubularin related protein 1	59.0905	84.5968	12.8538	227.721	309.784	287.219
A_33_P3258660	SCD5	stearoyl-CoA desaturase 5	61.0873	216.987	83.6013	557.501	776.06	766.469
A_33_P3278868	HEATR5A	HEAT repeat containing 5A	335.364	161.487	214.17	1379.99	705.378	3554.3
A_24_P927716	SLED1	proteoglycan 3, pro eosinophil major basic protein 2 pseudogene	4.82711	5.25341	3.83823	17.8571	59.9123	26.8524
A_21_P0014863	PPP2R5C	protein phosphatase 2 regulatory subunit B'gamma	229.26	305.409	261.726	1314.77	1804.61	2262.59
A_33_P3266828	TMEM8B	transmembrane protein 8B	261.29	273.247	344.548	1385.67	2389.77	2169.04
A_24_P288685	IL13RA1	interleukin 13 receptor subunit alpha 1	135.232	89.8114	61.97	509.122	602.691	699.054
A_23_P153320	ICAM1(CD54)	intercellular adhesion molecule 1	878.03	758.86	240.641	3839.84	2589.28	4455.57
A_21_P0004295	CDC42SE2	CDC42 small effector 2	5.88567	10.9404	5.42144	127.364	19.7263	38.1281
A_33_P3368695	HES7	hes family bHLH transcription factor 7	76.4086	54.5968	15.9254	278.434	343.579	189.809
A_33_P3246022			11.2698	12.952	6.16925	32.1742	156.984	48.4095
A_33_P3296499	PTPRK	protein tyrosine phosphatase, receptor type K	72.7368	176.599	103.342	485.813	1303.35	567.048
A_23_P1912	ZP1	zona pellucida glycoprotein 1	4.29754	11.239	4.33624	24.7706	65.6896	33.7196
A_23_P71727	CKS2	CDC28 protein kinase regulatory subunit 2	2276.28	5690.95	1006.42	9178.26	18943.3	19018.7
A_23_P39840	VAMP5	vesicle associated membrane protein 5	117.66	33.6202	96.9135	362.67	459.229	579.664
A_32_P10133	lnc-THNSL1-2		164.128	208.432	94.298	339.277	1479.37	1588.17
A_33_P3340828			11.2842	31.3949	8.1207	41.4558	147.084	116.508
A_33_P3349597			371.264	459.825	332.317	1874.2	1838.14	4059.02
A_23_P74737	EYA3	EYA transcriptional coactivator and phosphatase 3	19.4419	15.2226	15.2333	80.7004	93.3705	147.451
A_21_P0010942	FAM25A	family with sequence similarity 25 member A	56.1479	87.0817	123.184	356.075	678.2	606.727
A_23_P204436	GIT2	GIT ArfGAP 2	38.6883	99.4576	26.1005	416.49	143.971	404.744
A_33_P3278303			497.707	820.415	478.522	2700.98	2822.26	6173.02
A_33_P3306973			84.7454	143.938	53.5712	384.757	643.307	628.426
A_32_P34138	FAM25A	family with sequence similarity 25 member A	315.506	405.107	573.926	1913.7	3370.81	2700.98
A_23_P34396	RSRP1	arginine and serine rich protein 1	789.076	244.665	550.277	2115.59	3795.09	3106.72
A_23_P39237	ZFP36	ZFP36 ring finger protein	1349.73	682.864	1187.24	2821.33	10936.4	8312.29
A_23_P61466	CD163L1	CD163 molecule like 1	246.945	97.9677	109.81	670.424	641.68	1443.33
A_33_P3307955	ABCD3	ATP binding cassette subfamily D member 3	78.0094	52.0685	27.1389	357.456	171.074	421.195
A_23_P397238	FKBP1A	FKBP prolyl isomerase 1A	108.396	72.7219	37.9287	453.789	262.815	580.853
A_33_P3310415			6.0487	3.68919	3.61716	13.3811	58.2356	23.9522
A_21_P0000398	SNORD117	small nucleolar RNA, C/D box 117	9.06548	12.4352	16.2821	89.3613	119.655	38.989
A_24_P365679	RRP7B	ribosomal RNA processing 7 homolog B, pseudogene	10.2396	3.43126	7.73799	61.4675	19.754	50.8023
A_33_P3348872	PRDM16	PR/SET domain 16	41.1191	59.1845	36.98	193.103	337.348	312.932
A_33_P3349843			8.96939	13.4742	13.9131	28.0444	120.298	110.821
A_23_P217475	IDS	iduronate 2-sulfatase	963.073	806.499	465.665	3174.77	4983.98	5067.52
A_33_P3341787			61.1703	54.1359	21.5766	182.874	311.277	276.661
A_23_P156310	SKP2	S-phase kinase associated protein 2	84.121	121.319	106.678	528.491	697.322	640.197
A_24_P307974	TAF8	TATA-box binding protein associated factor 8	64.7902	158.455	47.5928	369.101	612.56	457.608
A_21_P0000234	SNORD38B	small nucleolar RNA, C/D box 38B	227.678	273.414	137.963	732.114	2696.69	911.421
A_21_P0000393	SNORD99	small nucleolar RNA, C/D box 99	194.019	126.431	87.705	459.393	1563.11	618.63
A_23_P67980	KLF7	Kruppel like factor 7	119.857	165.451	216.9	664.703	1869	706.732
A_24_P160401	CDCP1	CUB domain containing protein 1	198.191	261.52	235.24	581.179	2525.09	1646.58
A_33_P3347320	ADIRF-AS1	ADIRF antisense RNA 1	130.103	130.511	149.26	631.213	1398.97	564.773
A_23_P149050	LINC00115	long intergenic non-protein coding RNA 115	102.506	63.3294	89.2831	322.671	1246.18	282.04
A_23_P420196	SOCS1	suppressor of cytokine signaling 1	60.0823	28.1748	134.333	295.101	314.923	476.225
A_23_P201636	LAMC2	laminin subunit gamma 2	14614.3	10860.6	9864.04	61704.8	40361.5	120602
A_19_P00319080			26.3164	13.4974	28.1094	76.5684	139.283	178.341
A_33_P3289701	OR2T12	olfactory receptor family 2 subfamily T member 12	9.02512	5.8288	4.35991	25.157	26.3258	65.8883
A_33_P3589722	ATM	ATM serine/threonine kinase	173.946	61.7684	177.443	481.254	685.108	1085.88
A_23_P141894	PVR	poliovirus receptor	90.5114	65.7306	28.8995	360.889	260.906	337.869
A_24_P415624	MLXIP	MLX interacting protein	47.9632	100.7	75.5959	366.686	254.905	714.373
A_33_P3384885	ZNF511	zinc finger protein 511	42.6468	53.7075	50.2771	186.748	569.649	195.843
A_23_P211428	SMTN	smoothelin	49.5085	40.9374	53.3109	194.142	251.465	398.704
A_21_P0013999			24.3145	59.3623	27.0786	91.8152	303.87	251.348
A_33_P3337977	FIG4	FIG4 phosphoinositide 5-phosphatase	23.9905	23.9602	24.2573	96.947	200.01	126.397
A_33_P3388466	BTN3A1	butyrophilin subfamily 3 member A1	28.9643	24.5682	26.8136	114.365	102.16	281.142
A_33_P3351566	ETNK1	ethanolamine kinase 1	46.4553	15.3154	19.2285	81.4154	193.201	149.701
A_23_P256603	AFDN	afadin, adherens junction formation factor	43.1507	47.6918	14.7681	135.932	208.928	179.46
A_33_P3210916	SEC13	SEC13 homolog, nuclear pore and COPII coat complex component	6.0371	10.0989	5.99567	19.4345	60.4621	51.9916
A_33_P3239634	NFYA	nuclear transcription factor Y subunit alpha	42.9517	52.4262	55.2651	110.219	629.877	298.784
A_24_P350744	KHDC4	KH domain containing 4, pre-mRNA splicing factor	56.3923	56.5261	59.1056	134.669	776.295	295.368
A_33_P3376379	CNST	consortin, connexin sorting protein	126.431	97.3682	52.7945	263.961	891.508	447.439
A_21_P0000302	SNORA8	small nucleolar RNA, H/ACA box 8	477.573	369.44	253.208	1981.96	3083.73	1178.32
A_24_P359191	SLC6A6	solute carrier family 6 member 6	41.7775	91.2674	51.4056	146.837	411.004	517.219
A_33_P3331511	LINC00173	long intergenic non-protein coding RNA 173	69.3119	28.3044	33.0572	171.747	242.024	247.664
A_33_P3225268	QSOX1	quiescin sulfhydryl oxidase 1	259.272	230.305	153.677	2061	451.425	1564.15
A_33_P3313948	KLF11	Kruppel like factor 11	3.44423	13.7006	3.44998	19.6705	41.8905	30.0489
A_33_P3302245	TMEM59L	transmembrane protein 59 like	37.1586	19.6816	30.7792	189.555	217.253	82.1686
A_33_P3373765	DRD4	dopamine receptor D4	139.128	110.186	225.151	1090.1	526.977	895.672
A_33_P3214119			65.2368	24.14	15.0431	149.762	204.619	114.612
A_23_P56288	LENG8	leukocyte receptor cluster member 8	82.9891	60.149	52.1558	815.476	241.343	193.326
A_33_P3408529			4.66147	4.14951	4.99068	61.309	11.9305	19.067
A_33_P3231613	lnc-CSAG1-1		42.1193	14.3296	25.0221	158.997	141.616	95.8996
A_21_P0000453	SNORD115-37	small nucleolar RNA, C/D box 115-37	4.30834	6.66681	3.89427	12.8074	26.5276	46.9231
A_33_P3389178	RAPGEF3	Rap guanine nucleotide exchange factor 3	277.872	209.298	152.332	672.744	2130.43	875.113
A_33_P3330991	LOC100134237		51.6112	67.1946	35.5987	215.682	199.776	401.336
A_33_P3330877			4.39516	16.1315	4.04658	31.0624	43.5231	29.2588
A_33_P3258824	NOTCH2	notch receptor 2	2156.93	2334.02	684.552	7451.52	5249.69	12108.7
A_33_P3225273	QSOX1	quiescin sulfhydryl oxidase 1	82.6356	68.4057	63.3438	612.648	182.483	439.085
A_33_P3417751	C1orf61	chromosome 1 open reading frame 61	11.6406	10.5268	4.80135	23.5269	69.5776	49.2624
A_24_P182494	DUSP10	dual specificity phosphatase 10	197.276	142.248	74.5782	660.271	1015.51	424.509
A_33_P3264399			5.27163	10.5686	3.53119	24.5772	50.8062	21.3411
A_33_P3213468	ANKRD33B	ankyrin repeat domain 33B	24.6774	46.4455	18.0372	114.92	119.7	202.66
A_33_P3265956	CBFA2T2	CBFA2/RUNX1 translocation partner 2	792.876	465.471	358.235	2562.1	2945.73	2352.23

A_24_P352116	SNHG7	small nucleolar RNA host gene 7	109.229	242.345	70.7738	429.295	1178.7	487.93
A_21_P0001685	LOC102724481	long intergenic non-protein coding RNA 1702	11.6906	15.5247	10.5078	28.9686	118.529	71.1407
A_24_P285032	IDS	iduronate 2-sulfatase	81.0422	88.2076	46.8958	282.194	368.371	412.313
A_23_P119143	ICAM5	intercellular adhesion molecule 5	147.002	151.557	412.94	669.951	2033.91	844.458
A_19_P00806473			28.0626	42.7787	55.7293	187.766	441.836	99.1731
A_33_P3359781	CD99	CD99 molecule (Xg blood group)	3.91093	13.3045	4.85736	28.1485	31.0694	35.5265
A_33_P3339036	MECP2	methyl-CpG binding protein 2	755.156	568.596	640.197	2589.28	3458.14	3684.98
A_33_P3399263	IL15RA	interleukin 15 receptor subunit alpha	124.994	156.876	64.7154	589.941	287.446	893.162
A_33_P3265159	GAPVD1	GTPase activating protein and VPS9 domains 1	22.8107	9.55442	7.76361	37.766	86.9681	60.7836
A_21_P0012073			82.5248	70.7545	92.8748	268.906	543.656	431.306
A_33_P3350863	RETN	resistin	14.1189	16.6495	10.9048	32.9823	122.929	73.3787
A_23_P259292	C1QTNF5	C1q and TNF related 5	38.2791	58.2428	47.8235	150.15	302.116	266.797
A_21_P0012224			43.2874	58.9133	47.8454	161.39	312.168	271.444
A_33_P3486059			11.1432	5.8353	7.64042	35.318	43.9876	35.8279
A_33_P3324137	PRO0628	hypothetical protein LOC643837	198.077	149.171	95.2673	463.236	952.223	711.2
A_21_P0007160	LOC101928443		46.096	60.5118	58.8233	177.206	350.15	289.796
A_21_P0011816	IGSF3	immunoglobulin superfamily member 3	21.5177	20.807	26.5816	125.935	88.457	115.377
A_23_P53345	ARNTL2	aryl hydrocarbon receptor nuclear translocator like 2	537.939	159.927	268.028	1296.25	822.742	2289.54
A_33_P3325120	SOX13	SRY-box 13	32.5756	32.9034	85.0997	223.247	171.693	249.287
A_33_P3240843	TMEM71	transmembrane protein 71	75.1125	124.883	81.3793	274.492	518.119	557.146
A_23_P336554	IL1RAP	interleukin 1 receptor accessory protein	126.156	108.489	89.6398	568.113	684.363	318.901
A_33_P3368900			76.8864	76.1708	58.4616	176.393	310.961	623.157
A_32_P213831	STRIP2	striatin interacting protein 2	79.557	64.9634	28.7047	234.577	294.998	212.981
A_33_P3208975	OR51S1	olfactory receptor family 51 subfamily S member 1	6.48126	5.01973	3.6083	11.5109	42.5726	23.4587
A_33_P3396492	CDH24	cadherin 24	90.8561	72.3175	30.5826	242.142	263.998	307.005
A_21_P0000173	C5orf56	chromosome 5 open reading frame 56	796.259	791.844	857.088	3278.41	2212.14	7183.55
A_21_P0002142	SPATA3-AS1	SPATA3 antisense RNA 1 (head to head)	244.665	319.405	310.784	970.533	1625.93	1469.62
A_23_P38618	PIGL	phosphatidylinositol glycan anchor biosynthesis class L	262.752	113.672	114.95	670.35	576.276	840.832
A_33_P3213408	OR10AD1	olfactory receptor family 10 subfamily AD member 1	10.838	17.5934	4.17645	42.9881	49.5026	35.3142
A_24_P158285	CYLC2	cylicin 2	4.64297	3.51872	3.52344	29.8308	10.6227	17.0535
A_21_P0002746			230.928	302.63	354.704	1058.15	1587.13	1366.33
A_33_P3232498	PCARE	photoreceptor cilium actin regulator	5.7372	6.07089	9.55769	15.2839	32.8487	61.2884
A_24_P206604	PFKFB3	6-phosphofructo-2-kinase/fructose-2,6-biphosphatase 3	29.8232	23.226	21.3364	80.3653	137.525	122.929
A_33_P3254666	LZTS2	leucine zipper tumor suppressor 2	227.892	240.036	345.326	610.054	1834.54	1531.32
A_21_P0000360	SNORA77	small nucleolar RNA, H/ACA box 77	83.2089	109.399	127.992	330.408	643.863	492.489
A_33_P3243399	SLMAP	sarcolemma associated protein	96.043	43.8114	56.4451	192.724	461.28	240.163
A_33_P3242663	VASH2	vasohibin 2	14.0162	15.3346	10.8897	28.9043	107.996	67.2892
A_33_P3315074			46.1716	63.9428	36.2111	175.671	178.222	304.983
A_21_P0010339			8.79132	19.0017	8.66842	27.1678	84.9986	55.8613
A_24_P208081	PLA2G2F	phospholipase A2 group IIF	29.698	50.232	34.4843	99.7526	227.096	202.065
A_33_P3291708			32.4602	45.8158	34.3221	117.501	192.252	200.48
A_24_P67898	OGA	O-GlcNAcase	523.798	157.193	169.086	1505.86	1040.12	786.471
A_33_P3362239			136.624	148.685	104.796	468.476	462.286	863.409
A_33_P3212645	NOTCH2NL	notch 2 N-terminal like A	6804	6652.01	2296.33	20517.4	13846.8	31861.9
A_21_P0003367	LOC101928785		4.11858	3.42227	6.99109	19.1907	15.2034	28.7027
A_23_P15394	CD68	CD68 molecule	210.949	577.387	348.295	1035.36	1268.1	2739.16
A_23_P408768	DOT1L	DOT1 like histone lysine methyltransferase	43.399	68.3245	44.6206	133.86	273.389	305.868
A_21_P0009972	LINC01273	long intergenic non-protein coding RNA 1273	125.646	79.5453	41.9911	320.947	402.482	271.791
A_24_P556318	SAA3P	serum amyloid A3, pseudogene	3.70348	6.85683	6.29881	34.6701	15.4137	24.9977
A_21_P0014256	STK32C	serine/threonine kinase 32C	858.105	852.049	994.669	3705.4	4203.51	3894.93
A_23_P93658	ASIC3	acid sensing ion channel subunit 3	6.95259	16.4868	12.8996	30.8416	59.4804	66.141
A_33_P3268129	LINC00862	long intergenic non-protein coding RNA 862	5.74005	3.97178	4.2683	26.1928	18.5868	16.1382
A_23_P200801	PDE4DIP	phosphodiesterase 4D interacting protein	93.4468	90.7779	75.2664	241.304	324.07	655.25
A_23_P156732	PHF1	PHD finger protein 1	323.258	467.316	383.014	1271.97	1906.52	1911.22
A_33_P3385656	FNDC9	fibronectin type III domain containing 9	26.6829	23.5525	34.4388	70.4772	181.379	135.475
A_21_P0001492	LINC01343	long intergenic non-protein coding RNA 1343	16.0295	13.2655	18.6067	73.9765	72.5797	58.0463
A_24_P119259	BLZF1	basic leucine zipper nuclear factor 1	24.7288	18.9525	20.8848	42.3039	153.817	116.727
A_21_P0012143			9.93136	13.1873	11.162	39.9043	101.996	27.7992
A_23_P162010	CCKBR	cholecystokinin B receptor	4.67143	3.72512	7.54658	24.4864	19.9285	20.6936
A_21_P0009321	LOC101929494		65.0318	86.1221	39.0608	240.366	250.627	276.076
A_33_P3262012			562.877	1289.52	545.318	1859.59	4919.52	3258.11
A_33_P3396972			27.5101	34.4049	32.7809	85.5173	163.343	166.7
A_33_P3326730	CCDC183	coiled-coil domain containing 183	13.9144	7.02593	6.63485	32.6706	27.4468	54.2201
A_23_P23616	PLEKHN1	pleckstrin homology domain containing N1	94.8871	120.041	39.401	301.243	426.318	259.727
A_23_P383819	TBX3	T-box 3	21.3133	33.9123	11.496	62.1418	98.4582	100.649
A_33_P3259933	lnc-RNF208-1		3.723	8.2105	3.98705	26.7422	15.4657	21.653
A_33_P3267280	CTDSP2	CTD small phosphatase 2	188.404	145.851	96.947	683.469	680.871	418.752
A_21_P0000333	SNORA46	small nucleolar RNA, H/ACA box 46	33.9123	38.1617	28.8279	158.055	213.801	80.719
A_23_P209394	CFLAR	CASP8 and FADD like apoptosis regulator	271.21	142.53	174.631	870.721	501.252	1129.37
A_33_P3342628	HES4	hes family bHLH transcription factor 4	12242.4	8963.68	3269.16	27657	31251	30049.8
A_33_P3311439	GCH1	GTP cyclohydrolase 1	47.1838	110.394	52.808	222.795	265.497	336.224
A_24_P290354	ZFAND5	zinc finger AN1-type containing 5	1672.97	1532.23	1741.59	6029.37	6752.79	7860.02
A_33_P3384667	DCAF5	DDB1 and CUL4 associated factor 5	27.7194	47.2922	24.5415	80.9359	210.098	135.232
A_33_P3231908	POU3F3	POU class 3 homeobox 3	3.56001	5.92504	8.32139	22.9544	14.4187	37.6055
A_23_P350555	TCP10L	t-complex 10 like	14.3802	11.336	26.3164	83.7571	77.4974	46.787
A_32_P205944	RGPD5	RANBP2-like and GRIP domain containing 5	868.749	360.675	238.147	2200.05	1581.57	1516.64
A_33_P3289050			11.3284	11.7026	8.68374	23.2324	48.5401	72.0208
A_23_P377245	LOC653160	uncharacterized LOC653160	40.5996	18.3931	28.7341	108.997	107.914	127.046
A_33_P3320718	C16orf13	methyltransferase like 26	237.738	238.49	251.348	610.681	1620.44	1001
A_33_P3404014	FAM209B	family with sequence similarity 209 member B	7.34541	4.38368	3.53879	14.6296	30.0104	17.8795
A_33_P3407636	YWHAE	tyrosine 3-monooxygenase/tryptophan 5-monooxygenase activation protein epsilon	50.2876	28.7266	33.7955	104.214	174.473	184.132
A_21_P0003488	LOC101929491	uncharacterized LOC101929491	15.55	7.18138	5.0016	42.2658	32.3027	27.714
A_33_P3367392	FAM167B	family with sequence similarity 167 member B	104.594	97.5541	95.3175	308.933	468.009	443.211
A_23_P96542	VMA21	vacuolar ATPase assembly factor VMA21	2008.27	3139.66	1562.05	5811.6	13644.8	8174.19
A_33_P3280916	SNHG4	small nucleolar RNA host gene 4	6.99697	13.9759	11.0953	26.4539	74.1684	36.3624
A_33_P3297217	NAAA	N-acylethanolamine acid amidase	161.545	82.5857	125.975	576.276	275.796	689.489
A_33_P3344292	SAMD4A	sterile alpha motif domain containing 4A	11.1409	6.84855	7.96935	20.9765	48.5797	38.8205
A_23_P159956	MID2	midline 2	292.564	200.055	166.496	1011.25	688.882	906.77
A_33_P3289661	OCR1	ovarian cancer-related protein 1	7.85052	4.78735	7.53095	24.5811	36.6747	20.3482
A_33_P3282963			26.1928	10.5378	19.4776	58.7298	80.495	73.4096
A_33_P3671378	CERCAM	cerebral endothelial cell adhesion molecule	229.026	162.777	85.4885	719.038	453.092	629.62
A_33_P3231472	TOR1B	torsin family 1 member B	95.9389	78.2301	81.1	193.218	374.617	537.939
A_33_P3314559	RAB3C	RAB3C, member RAS oncogene family	6.94479	9.71715	12.2494	31.6913	51.0821	32.6132
A_33_P3330473	RBM41	RNA binding motif protein 41	44.0177	30.0922	37.8713	114.575	157.051	173.173
A_32_P231617	TM4SF1	transmembrane 4 L six family member 1	476.379	235.24	167.389	1286.7	883.747	1020.71
A_24_P384029			9.74457	16.8031	8.64038	37.817	39.5161	57.841
A_23_P129188	CALML4	calmodulin like 4	187.766	96.3899	102.845	359.684	724.592	436.115
A_33_P3317442	MCF2L	MCF.2 cell line derived transforming sequence like	11.1358	28.875	16.0773	87.8777	71.5227	50.0231
A_33_P3313846	ZDHHC8	zinc finger DHHC-type containing 8	60.1654	99.1377	93.9032	199.515	415.871	408.904
A_33_P3274930			5.49126	7.13384	8.74266	22.9723	20.0518	44.7268
A_24_P147398	VPS53	VPS53 subunit of GARP complex	18.2885	16.0966	9.18092	69.1341	39.2551	59.6903

A_24_P186943	ELN	elastin	60.0076	61.0396	64.4775	140.748	320.113	313.774
A_23_P259580	TAPBP	TAP binding protein	92.327	111.21	59.6406	278.365	376.959	348.372
A_24_P305038	CRHR1-IT1	long intergenic non-protein coding RNA	49.166	80.6806	42.3914	128.065	379.116	206.457
A_33_P3355252			5.47087	11.2167	4.355	19.5061	21.138	37.817
A_24_P358305			38.1396	44.5626	15.6392	107.683	106.299	134.618
A_33_P3233150	ZSWIM4	zinc finger SWIM-type containing 4	119.274	101.669	120.648	377.958	292.214	762.785
A_21_P0014355	LOC100134391	uncharacterized LOC100134391	57.4314	36.3212	56.5261	108.47	194.417	317.25
A_23_P342053	RBBP6	RB binding protein 6, ubiquitin ligase	59.7859	78.8617	69.8959	146.198	419.292	298.063
A_33_P3274245	ENDOV	endonuclease V	53.1596	76.7695	71.8811	185.873	305.868	279.676
A_21_P0012869	LOC728554	THO complex 3 pseudogene	26.0041	35.2851	15.5267	94.1917	59.3998	136.89
A_33_P3286387	EHMT1	euchromatic histone lysine methyltransferase 1	50.4151	100.396	38.0603	176.1	222.109	263.601
A_21_P0007476	LINC00592	long intergenic non-protein coding RNA 592	403.592	461.219	186.781	1525.55	1194.65	1016.53
A_23_P134851	DOK2	docking protein 2	6.35313	5.05727	3.71822	24.2299	19.4419	13.4323
A_33_P3266923	LMTK2	lemur tyrosine kinase 2	295.975	181.379	129.229	638.78	667.948	860.714
A_23_P35205	RCAN3	RCAN family member 3	153.817	152.447	133.677	506.041	515.902	633.32
A_24_P915692	PHLDA1	pleckstrin homology like domain family A member 1	250.561	264.272	145.372	710.732	603.633	1176.35
A_23_P97064	FBXO6	F-box protein 6	78.1899	88.1365	85.9982	303.771	306.882	331.219
A_23_P25994	LGMN	legumain	221.957	272.148	144.337	1016.69	440.408	1006.7
A_33_P3423941	IFITM1	interferon induced transmembrane protein 1	2720.86	1096.13	2397.63	7752.24	5757.17	8225.67
A_33_P3282252			47.6818	28.5397	37.7991	129.487	160.192	126.468
A_33_P3257553	CNTRL	centriolin	12.5966	10.7821	10.1224	27.5346	59.1705	42.6075
A_33_P3223898	PRSS45	protease, serine 45	7.5113	12.9598	5.31736	22.0123	41.9911	28.215
A_33_P3376873			78.9076	84.4592	33.9214	320.603	181.074	195.056
A_33_P3366241	SCARA5	scavenger receptor class A member 5	18.9891	15.1612	11.4972	32.4602	79.2443	64.2645
A_32_P222961	SPIN4	spindlin family member 4	180.751	170.909	106.097	366.769	705.546	630.203
A_21_P0004050			5.03489	8.15191	6.98531	18.5057	17.0125	44.8418
A_23_P31532	ZC3HAV1L	zinc finger CCCH-type containing, antiviral 1 like	333.702	443	479.262	1069.01	1952.97	1662.67
A_24_P55148	HIST1H2BJ	histone cluster 1 H2B family member j	306.071	324.559	310.043	1310.04	930.71	1234.31
A_33_P3293753	KRTAP10-9	keratin associated protein 10-9	31.2799	39.5759	36.9521	88.9214	143.537	173.481
A_23_P118427	MAP2K3	mitogen-activated protein kinase kinase 3	315.74	335.94	129.099	1040.49	675.452	923
A_21_P0003212			446.625	419.919	240.684	1019.96	2016.92	1023.7
A_33_P3231247			9.14072	15.6987	8.30033	42.4511	34.8885	37.1124
A_33_P3229067	HIST1H2BN	histone cluster 1 H2B family member n	187.898	171.028	275.468	832.524	626.307	768.369
A_23_P24966	USP2	ubiquitin specific peptidase 2	5.44971	7.42357	5.30041	14.0886	30.0691	22.8167
A_21_P0009290			28.4219	28.7309	30.7846	64.2603	154.99	113.323
A_24_P257108	TANK	TRAF family member associated NFKB activator	73.2343	76.6219	85.435	187.898	451.716	252.893
A_23_P62607	IL22RA1	interleukin 22 receptor subunit alpha 1	132.224	174.032	101.044	663.481	312.645	500.091
A_24_P328504	SP140	SP140 nuclear body protein	153.767	189.555	138.484	365.854	671.667	725.066
A_33_P3248245			14.7884	18.5868	15.9294	50.0366	76.1871	50.5864
A_33_P3401428	TMEM38B	transmembrane protein 38B	74.5782	132.394	63.0255	223.456	285.624	429.383
A_33_P3278211	MMRN2	multimerin 2	49.7449	42.8735	45.0218	112.602	220.534	167.705
A_33_P3337540	IFNAR2	interferon alpha and beta receptor subunit 2	507.95	437.395	338.687	972.421	1828.87	1828.29
A_21_P0013492	LOC101929488	uncharacterized LOC101929488	16.8126	27.4216	22.846	46.3015	101.768	95.7279
A_21_P0002014			4.51579	3.61286	3.61065	18.1357	9.38311	14.7167
A_23_P19291	TUBB2A	tubulin beta 2A class IIa	2310.92	1134.89	1131.86	5738.38	4295.44	5111.6
A_24_P201153	TJP2	tight junction protein 2	144.221	150.406	69.5489	534.058	355.848	336.569
A_33_P3226228			25.9549	25.114	16.2218	113.461	64.0987	61.405
A_23_P428184	HIST1H2AD	histone cluster 1 H2A family member d	3795.09	2104.2	4646.09	10709	8981.26	16282.7
A_24_P207479	DEDD2	death effector domain containing 2	1323.79	1209.58	1317.09	3253.89	8409.62	3251.19
A_33_P3263412			26.0423	29.2899	19.1354	70.9292	99.3935	86.7537
A_33_P3390057	TM4SF1	transmembrane 4 L six family member 1	2031.94	840.532	903.164	4998.59	3640.35	3492.87
A_33_P3363316	LINC00957	long intergenic non-protein coding RNA 957	14.4291	15.0006	15.5938	29.311	75.6579	62.6648
A_33_P3341189	UBE2A	ubiquitin conjugating enzyme E2 A	2702.46	2140.98	1177.43	6500.47	7177.39	5999.79
A_33_P3353275			3.65358	6.14582	9.38748	25.8411	16.0473	20.8788
A_21_P0012547			11.854	18.094	11.142	31.6468	50.7157	60.8975
A_33_P3405103	FLYWCH1	FLYWCH-type zinc finger 1	143.571	194.231	210.822	454.4	618.799	850.987
A_33_P3377763	FLJ36000	uncharacterized FLJ36000	66.4846	51.6369	53.4346	138.577	356.106	151.284
A_33_P3364836			12.6913	9.49391	7.24431	30.4108	24.3103	47.8265
A_33_P3265950	CBFA2T2	CBFA2/RUNX1 translocation partner 2	168.515	93.9032	109.205	393.439	424.771	417.659
A_23_P358944	PML	promyelocytic leukemia	2604.73	2712.39	1700.97	8365.79	6572.95	8753.6
A_23_P218369	CCL14	C-C motif chemokine ligand 14	6.39778	11.5824	8.49833	21.4948	32.5795	35.1819
A_23_P63402	GPSM2	G protein signaling modulator 2	289.604	331.809	280.474	886.924	805.596	1458.04
A_32_P64200	GUCA1B	guanylate cyclase activator 1B	13.154	13.2608	20.2333	47.4716	62.7609	44.7884
A_21_P0003279	lnc-WNT7A-1		13.0816	13.5203	12.2753	40.7415	54.6687	36.4202
A_33_P3272169	CLIP2	CAP-Gly domain containing linker protein 2	45.9161	49.8941	30.959	128.605	146.872	139.439
A_33_P3375681	LOC100129027	uncharacterized LOC100129027	10.6242	12.05	13.735	27.5101	45.1047	52.42
A_21_P0004584			7.27909	9.30388	9.6325	20.9314	31.162	36.939
A_21_P0013515			8.9176	7.31642	3.45849	23.3494	19.2498	18.5387
A_33_P3419945			68.8838	54.1495	143.289	250.772	295.508	265.726
A_33_P3361457	IFNAR2	interferon alpha and beta receptor subunit 2	214.948	243.617	141.069	421.235	883.324	726.655
A_33_P3361417	C6orf57	succinate dehydrogenase complex assembly factor 4	367.327	245.181	385.045	797.121	1213.93	1302.04
A_24_P353905	MXRA8	matrix-remodelling associated 8	21.6127	22.9638	15.549	39.1356	80.5665	88.2823
A_19_P00320407	LOC100506047	long intergenic non-protein coding RNA 2580	33.6616	16.8555	20.9733	76.8442	79.6537	67.9565
A_33_P3408203	TGFA	transforming growth factor alpha	429.2	269.85	287.577	861.835	859.241	1570.65
A_33_P3277097	LY6G6E	lymphocyte antigen 6 family member G6E	40.9885	64.7387	64.2799	114.151	241.138	212.652
A_23_P85543	RNF2	ring finger protein 2	227.523	181.695	180.324	564.442	668.087	673.953
A_21_P0004143	LINC01335	long intergenic non-protein coding RNA 1335	11.7822	10.5663	9.40896	22.3236	38.2847	45.8807
A_33_P3258013	UBE3B	ubiquitin protein ligase E3B	1294.08	2013.42	1054.12	4326.44	4369.98	4813.02
A_33_P3683076	MAGED2	MAGE family member D2	87.2568	72.9595	108.267	277.362	193.458	424.857
A_33_P3254634	PDIA5	protein disulfide isomerase family A member 5	170.333	145.969	81.7226	299.456	481.107	462.237
A_19_P00802154	DST	dystonin	38.7924	27.4723	43.2032	72.7368	133.078	153.767
A_24_P323545	MYH14	myosin heavy chain 14	48.1336	27.4444	40.6883	98.9445	146.255	120.006
A_33_P3415345	ALS2	alsin Rho guanine nucleotide exchange factor ALS2	39.9043	42.4445	33.3243	88.096	135.493	151.529
A_32_P59811			49.5549	44.4937	57.5504	101.996	228.55	171.206
A_23_P62115	TIMP1	TIMP metallopeptidase inhibitor 1	1613.09	1309.43	2156.93	3709.94	6778.51	5688.59
A_33_P3436316	ASXL1	ASXL transcriptional regulator 1	531.099	429.2	432.28	974.543	1458.36	2176.87
A_33_P3343155	GNAQ	G protein subunit alpha q	132.394	138.926	208.369	392.761	628.684	487.305
A_21_P0007140	lnc-PGAP2-1	lnc-PGAP2-1	10.715	9.90767	6.68984	25.7737	23.8115	35.9247
A_21_P0006228	lnc-STXBP1-1	lnc-STXBP1-1	18.679	23.4004	12.2363	52.6769	46.4955	67.1685
A_21_P0013062	LOC101928106	long intergenic non-protein coding RNA 2522	20.9705	15.6093	11.8664	41.2674	56.7484	50.9886
A_21_P0005973	LOC101927459	long intergenic non-protein coding RNA 1608	4.63669	4.83441	5.91645	15.9108	17.4169	14.6376
A_32_P178945	YOD1	YOD1 deubiquitinase	410.071	516.6	696.774	1238.54	2160.02	1681.19
A_23_P207774	KSR1	kinase suppressor of ras 1	17.0096	17.4514	12.0256	33.4779	59.324	54.3384
A_33_P3417487	SCUBE1	signal peptide, CUB domain and EGF like domain containing 1	22.1923	31.7534	27.4723	62.1711	91.5055	101.889
A_33_P3325914	TAPBP	TAP binding protein	481.613	597.621	324.402	1385.97	1250.34	1570.05
A_33_P3227258			13.1743	14.4886	17.8147	36.6654	52.216	51.4876
A_23_P20122	ZC3HAV1	zinc finger CCCH-type containing, antiviral 1	39.4199	63.782	39.7839	162.443	129.207	138.134
A_33_P3394213	GRIN3B	glutamate ionotropic receptor NMDA type subunit 3B	4.60536	3.49631	3.54901	17.6245	9.19366	9.99407
A_23_P405873	C9orf72	C9orf72-SMCR8 complex subunit	23.2426	24.7922	24.4952	49.2444	94.5619	83.9441
A_33_P3264780	CDK8	cyclin dependent kinase 8	60.9304	83.7078	52.1711	145.783	281.668	175.917
A_33_P3258265	SEMA6C	sema domain, transmembrane domain (TM), and cytoplasmic domain, (semaphorin) 6C	38.0255	47.1601	27.3728	80.881	136.385	120.743

A_23_P46604	NIPAL3	NIPA like domain containing 3 [Homo sapiens	265.094	209.474	199.177	795.626	433.994	858.105
A_21_P0001246	LOC101927851	uncharacterized LOC101927851	157.342	156.071	298.619	545.446	564.442	629.235
A_23_P502832	RBM12	RNA binding motif protein 12	185.098	142.067	170.283	375.628	589.941	526.327
A_33_P3286302	TNFRSF14	TNF receptor superfamily member 14	79.2912	94.8105	100.582	317.25	261.52	235.988
A_33_P3225418	HCG9	HLA complex group 9	5.97476	8.60193	9.68659	18.8909	32.1029	21.0817
A_33_P3349414	ZBTB43	zinc finger and BTB domain containing 43	823.691	900.093	1184.06	1996.61	3123.17	3608.69
A_24_P50801	NRP2	neuropilin 2	14.8544	26.6982	16.761	51.2619	50.0146	66.3879
A_23_P41365	SMR3A	submaxillary gland androgen regulated protein 3A	25.6104	35.5265	36.1578	72.7809	100.118	114.781
A_23_P147109	TOR4A	torsin family 4 member A	397.941	459.966	241.091	929.351	1184.62	1016.69
A_33_P3325395			14.5631	15.3395	10.6098	31.2329	44.2101	43.3631
A_21_P0000613	TFAP2A-AS1	TFAP2A antisense RNA 1	52.0264	55.4702	58.4044	165.393	114.389	224.072
A_33_P3219860	OPA1-AS1	OPA1 antisense RNA 1	17.2708	11.8685	11.9434	39.9488	42.8312	35.6888
A_19_P00803333	ZSWIM6	zinc finger SWIM-type containing 6	110.832	110.808	80.5337	203.771	336.533	348.675
A_23_P127663	PRRG4	proline rich and Gla domain 4	116.766	90.8016	104.356	407.728	232.561	280.474
A_24_P278172	ZFAND5	zinc finger AN1-type containing 5	397.195	334.779	531.492	1037.23	1250.84	1303.85
A_23_P163143	ACYP1	acylphosphatase 1	1231.73	1159.76	1380.71	2897.14	4709.47	3432.34
A_21_P0004859	BTN2A1	butyrophilin subfamily 2 member A1	54.3384	52.5018	50.7737	111.763	206.682	148.685
A_23_P58293	UBE2D3	ubiquitin conjugating enzyme E2 D3	1879.48	1766.25	1299.05	4910.44	3772.79	5492.55
A_33_P3243394	SLMAP	sarcolemma associated protein	21.0279	16.2381	17.7263	39.6649	61.4775	58.5429
A_21_P0014778	LOC100507516	uncharacterized LOC100507516	11.7708	10.066	11.6645	29.6388	23.7313	46.2545
A_33_P3371954	IGSF8	immunoglobulin superfamily member 8	126.704	110.832	145.45	276.222	468.476	368.243
A_24_P192988	CCDC89	coiled-coil domain containing 89	8.67266	9.0244	10.1883	21.2587	27.8482	31.1768
A_23_P79331	TMBIM1	transmembrane BAX inhibitor motif containing 1	1609.26	2570.89	1666.76	5719.67	6683.25	4171.9
A_32_P50123	SRGAP2B	SLIT-ROBO Rho GTPase activating protein 2B	52.7193	61.8848	50.0707	149.701	169.292	146.698
A_19_P00315967			45.9098	34.5705	56.4578	139.36	111.763	128.046
A_32_P440768	ALOX12P2	ALOX12P2 arachidonate 12-lipoxygenase pseudogene 2	90.1062	107.89	91.3898	257.422	307.834	247.943
A_33_P3238425	SLC2A7	solute carrier family 2 member 7	13.4941	7.49568	7.76082	24.5112	27.233	25.9634
A_33_P3415748	LOC100129072	uncharacterized LOC100129072	11.9263	18.7989	12.1822	31.8472	41.2347	45.7842
A_23_P137046	NYX	nyctalopin	276.894	280.368	379.116	724.795	1119.48	792.028
A_23_P147826	RAP2C	RAP2C, member of RAS oncogene family	100.224	88.626	62.4414	179.46	270.741	248.781
A_19_P00322673			12.0469	13.3247	12.7516	27.4802	34.2986	46.5189
A_21_P0004860	BTN2A1	butyrophilin subfamily 2 member A1	110.658	109.037	109.115	230.928	395.044	307.194
A_33_P3370364	PRLHR	prolactin releasing hormone receptor	177.405	215.206	193.687	716.506	435.418	501.943
A_33_P3308101			152.2	174.859	190.663	366.165	515.735	568.48
A_32_P102062	FOXO3	forkhead box O3	156.071	141.069	158.84	347.451	586.797	361.879
A_33_P3400292			11.6363	12.3037	13.2928	27.4238	45.2471	32.2121
A_23_P34942	EVI5	ecotropic viral integration site 5	24.9787	17.5996	17.9817	52.5643	56.6441	55.2859
A_33_P3265376	HOMER3	homer scaffold protein 3	255.297	171.206	226.248	571.986	710.277	501.14
A_23_P310350	SHPK	sedoheptulokinase	295.508	368.461	283.812	639.116	1028.62	956.836
A_33_P3295917	IL7	interleukin 7	7.50421	7.19988	8.06244	20.7573	20.9475	20.3343
A_33_P3238074	MIEF1	mitochondrial elongation factor 1	17149.3	17664.2	19018.7	47544.9	44705.3	54132.9
A_21_P0000126	TGFBR3L	transforming growth factor beta receptor 3 like	155.208	171.799	149.604	400.836	515.318	367.864
A_23_P137423	IGSF8	immunoglobulin superfamily member 8	261.82	227.204	311.741	599.126	840.832	698.432
A_33_P3246885	DMKN	dermokine	1413.14	1288.35	1318.96	2668.02	4257.56	3906.8
A_33_P3317211	MECP2	methyl-CpG binding protein 2	50.8164	43.2396	51.8197	97.1282	139.524	154.543
A_23_P259413	CDV3	CDV3 homolog	3033.1	1909.75	2283.27	5731.92	6136.83	6752.79
A_23_P132226	TPST2	tyrosylprotein sulfotransferase 2	605.852	744.419	852.049	1630.11	2151.2	1908.94
A_33_P3279984			6.49108	9.95195	7.90781	19.4208	21.8728	20.4002
A_33_P3232038	RBAK-RBAKDN	RBAK-RBAKDN readthrough	261.605	267.078	265.226	551.36	772.788	728.678
A_21_P0007042	LOC101927946	uncharacterized LOC101927946	8.37467	8.1286	8.82206	16.9186	24.4751	24.2573
A_24_P99216	LRP10	LDL receptor related protein 10	599.126	524.335	680.232	1584.9	1431.75	1555.44
